# Hybrid Quinoline-Thiosemicarbazone Therapeutics as a New Treatment Opportunity for Alzheimer’s Disease‒Synthesis, In Vitro Cholinesterase Inhibitory Potential and Computational Modeling Analysis

**DOI:** 10.3390/molecules26216573

**Published:** 2021-10-30

**Authors:** Sumera Zaib, Rubina Munir, Muhammad Tayyab Younas, Naghmana Kausar, Aliya Ibrar, Sehar Aqsa, Noorma Shahid, Tahira Tasneem Asif, Hashem O. Alsaab, Imtiaz Khan

**Affiliations:** 1 Department of Biochemistry, Faculty of Life Sciences, University of Central Punjab, Lahore 54590, Pakistan; muhammadtayyabyounassst@gmail.com; 2 Department of Chemistry, Kinnaird College for Women, Lahore 54000, Pakistan; w14bche002@gmail.com (S.A.); noormashahid@gmail.com (N.S.); tahiratasneem1998@gmail.com (T.T.A.); 3 Department of Chemistry, University of Gujrat, Gujrat 50700, Pakistan; naghmana.kousar@uog.edu.pk; 4 Department of Chemistry, Faculty of Natural Sciences, The University of Haripur, Haripur 22620, Pakistan; aliya.ibrar@uoh.edu.pk; 5 Department of Pharmaceutics and Pharmaceutical Technology, Taif University, P.O. Box 11099, Taif 21944, Saudi Arabia; h.alsaab@tu.edu.sa; 6 Department of Chemistry, Manchester Institute of Biotechnology, The University of Manchester, 131 Princess Street, Manchester M1 7DN, UK

**Keywords:** quinoline, thiosemicarbazone, molecular design, hybridization, Alzheimer’s disease, neurodegeneration, drug therapy, cholinesterases, enzyme inhibition, molecular docking

## Abstract

Alzheimer’s disease (AD) is a progressive neurodegenerative disorder and the leading cause of dementia worldwide. The limited pharmacological approaches based on cholinesterase inhibitors only provide symptomatic relief to AD patients. Moreover, the adverse side effects such as nausea, vomiting, loss of appetite, muscle cramps, and headaches associated with these drugs and numerous clinical trial failures present substantial limitations on the use of medications and call for a detailed insight of disease heterogeneity and development of preventive and multifactorial therapeutic strategies on urgent basis. In this context, we herein report a series of quinoline-thiosemicarbazone hybrid therapeutics as selective and potent inhibitors of cholinesterases. A facile multistep synthetic approach was utilized to generate target structures bearing multiple sites for chemical modifications and establishing drug-receptor interactions. The structures of all the synthesized compounds were fully established using readily available spectroscopic techniques (FTIR, ^1^H- and ^13^C-NMR). In vitro inhibitory results revealed compound **5b** as a promising and lead inhibitor with an IC_50_ value of 0.12 ± 0.02 μM, a 5-fold higher potency than standard drug (galantamine; IC_50_ = 0.62 ± 0.01 μM). The synergistic effect of electron-rich (methoxy) group and ethylmorpholine moiety in quinoline-thiosemicarbazone conjugates contributes significantly in improving the inhibition level. Molecular docking analysis revealed various vital interactions of potent compounds with amino acid residues and reinforced the in vitro results. Kinetics experiments revealed the competitive mode of inhibition while ADME properties favored the translation of identified inhibitors into safe and promising drug candidates for pre-clinical testing. Collectively, inhibitory activity data and results from key physicochemical properties merit further research to ensure the design and development of safe and high-quality drug candidates for Alzheimer’s disease.

## 1. Introduction

Alzheimer’s disease (AD), a chronic neurodegenerative disorder, is the leading cause of senile dementia. The typical symptoms include the memory dysfunction, cognitive impairment, psychiatric and behavioral abnormality, and difficulty in performing everyday tasks [1,2,3]. This multifaceted neurodegenerative disorder is one of the leading causes of death in elderly people and continues to be a social, health and economic burden on society. The exact molecular mechanism for the pathogenesis of AD is not well-understood yet; however, several hypotheses have been proposed explaining the initiation of neurodegeneration in Alzheimer’s disease. These include cholinergic hypothesis (pathological changes and the dysfunction of the neuro-cholinergic system), amyloid hypothesis (β-amyloid tangles and aggregations inducing neural apoptosis, tau protein hyperphosphorylation forming senile plaque), oxidative stress hypothesis (neuro-inflammation and increasing level of reactive oxygen radicals), and bio-metal hypothesis (deregulation of transition bio-metals in AD patients). Among these, the design and development of new and potent inhibitors based on central cholinergic hypothesis remains the most common and clinically tested strategy for AD therapy [4,5,6,7,8].

Cholinesterase (ChE), namely acetylcholinesterase (AChE, EC 3.1.1.7) and butyrylcholinesterase (BuChE, EC 3.1.1.8), catalyze the hydrolysis of cholinergic neurotransmitters. Acetylcholine (ACh) is predominantly decomposed by AChE compared to BuChE, thus the inhibition of AChE to increase the level of ACh remains a promising strategy for the treatment of Alzheimer’s disease [9,10]. The crystal structure of this enzyme reveals the presence of a catalytic active site (CAS) and a peripheral anionic site (PAS) linked through a 20 Å long gorge. Furthermore, the role of AChE in the induction of AD through the pro-aggregation activity of the Aβ protein, formation of reactive oxygen species (ROS), calcium dysregulation, and neuronal dysfunction has been observed. Therefore, bioactive molecules with a potential to interact specifically with both catalytic site (PAS or CAS) residues can significantly help in the inhibition of AChE while eliminating Aβ aggregation [10].

The current treatment strategy provides only a symptomatic relief to AD patients. The previously approved (marketed) drugs, including tacrine, donepezil, rivastigmine, and galantamine, despite being diverse in structural features and pharmacokinetic profiles, are proving ineffective as medications in stopping or reversing the progression of AD [11]. Although, after 20 years, the approval of Aducanumab, treating the possible cause of the neurodegenerative disorder, rather than just the symptoms, provides a hope against this intractable condition [12], and the design of new and safer therapeutics to address multifactorial disease remains a promising research field. Therefore, employing a well-known pharmacophore hybridization strategy could prove effective in exerting a beneficial role in the treatment of AD [1,13].

**Figure 1:**
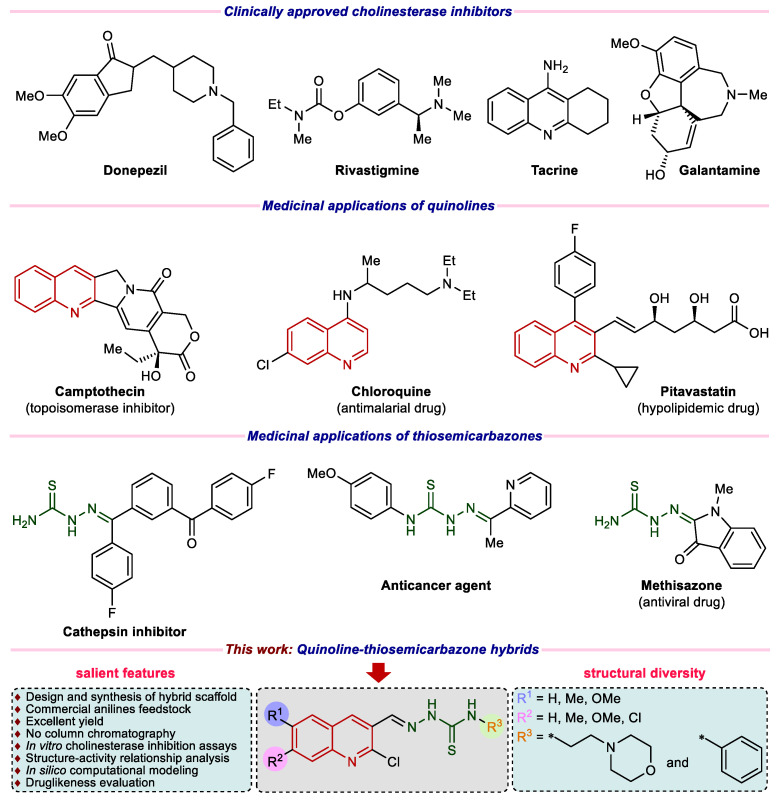
Examples of clinically approved drugs for AD and rationale of current study.

Quinoline (1-aza-naphthalene or benzo[b]pyridine) represents a class of nitrogen-containing heterocycles, which are well recognized for a diverse variety of pharmacological applications [14,15]. Various natural products, bioactive drug molecules, pharmaceuticals, and agrochemicals incorporate quinoline pharmacophore [16,17,18]. Notable medicinal applications associated with the quinoline heterocycle include anticancer, anti-tubercular, anti-proliferative, anti-malarial, antibacterial, anti-inflammatory, anti-protozoal, anti-fungal, anti-tumor, antioxidant, anti-HIV, anti-hypertensive, alkaline phosphatase inhibition and for the treatment of neurodegenerative disorders [19,20,21,22,23,24,25,26,27,28]. In parallel, thiosemicarbazones also display a wide plethora of biological properties ranging from anticancer, anti-bacterial, anti-tumor, anti-protozoal, anti-fungal, anti-leishmanial, and antiviral activities [29,30,31,32,33,34]. Thiosemicarbazone derivatives have also been employed as NDRG1 up-regulators, cathepsin inhibitors, and cholinesterase inhibitors for the treatment of Alzheimer’s disease [35,36,37,38]. Figure 1 represents illustrative examples of commercial drugs for AD therapy and importance of quinoline as well as thiosemicarbazone in medicinal/pharmaceutical chemistry.

Cognizant of the fact that both quinoline and thiosemicarbazone pharmacophores are promising candidates for the generation of molecular and structural libraries of anti-cholinesterase inhibitors, and taking inspiration from the tacrine scaffold that features a quinoline ring, we herein utilized a pharmacophore hybridization strategy to design and explore the wider chemical space for new and potent cholinesterase inhibitors with less side effects, extending on our previous efforts in the current area of research [39,40,41,42,43,44,45]. Furthermore, in view of high demand and omnipresence of nitrogen heterocycles in numerous drugs [46,47,48,49], we have introduced an additional morpholine ring of high therapeutic value [50,51,52,53,54] in the target structures to examine the effect on the cholinesterase inhibition (Figure 1). We have also calculated the ADME properties and the results were remarkably good.

## 2. Results and Discussion

### 2.1. Synthetic Chemistry

**Scheme 1:**
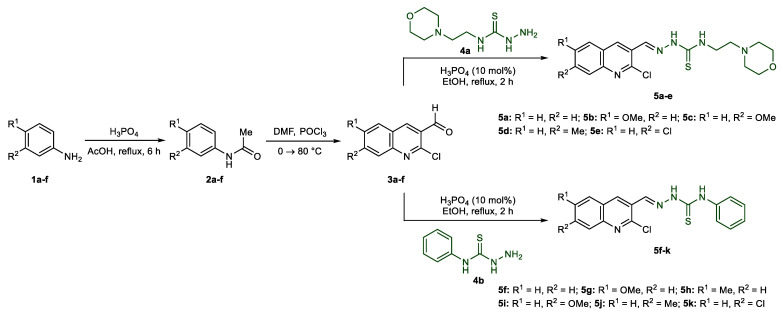
Synthetic route to quinoline-thiosemicarbazone hybrids **5a**–**k**.

The synthesis of quinoline-thiosemicarbazone hybrids **5a**–**k** was achieved using a facile and efficient multistep approach, as illustrated in Scheme 1. Several commercially available (un)substituted anilines were acetylated using orthophosphoric acid and acetic acid to afford acetanilides **2a**–**f** [45]. Subsequent Vilsmeier–Haack formylation using dimethylformamide and phosphoryl chloride provided 2-chloroquinoline-3-carbaldehydes **3a**–**f [55]**. Acid-catalyzed condensation of formylquinolines with commercially available *N*-(2-morpholinoethyl)hydrazinecarbothioamide **4a** and *N*-phenylhydrazinecarbothioamide **4b** gave the desired quinoline-thiosemicarbazone hybrids **5a**–**e** in excellent yields.

### 2.2. Spectroscopic Characterization

The condensation reaction of formylquinolines **3a**–**f** with thiosemicarbazides **4a** or **4b** affording quinoline-thiosemicarbazones **5a**–**k** was confirmed through ^1^H NMR spectroscopy where the target products feature a distinct singlet for azomethine (N=CH) proton (8.26–8.61 ppm). Two additional signals attributable to secondary thioamide protons, out of which =N-NH proton resonated at a relatively more downfield chemical shift as a singlet peak at 11.76–12.23 ppm compared to C-NH proton (10.15–10.29 ppm) also confirmed the structures of thiosemicarbazones **5f**–**k**. However, in case of compounds **5a**–**e**, C-NH proton displayed a comparatively upfield triplet signal (8.54–8.70 ppm) due to coupling with the adjacent methylene protons. Moreover, the disappearance of a very distinct aldehyde peak around 10 ppm also confirmed the consumption of **3a**–**f** during the course of reaction for the formation of target compounds **5a**–**k**.

The aromatic protons showed chemical shifts between 6.80 and 9.40 ppm according to their chemical environment, with H-4 of quinoline heterocycle appearing as the most deshielded proton. Moreover, in ^1^H NMR spectra of thiosemicarbazones **5a**–**e**, the morpholine ring protons gave two sets of signals around 2.45 and 3.60 ppm in addition to linear chain methylene protons that also exhibit a similar pattern.

The structures **5a**–**e** were further confirmed from ^13^C NMR data. The C=S carbon resonated at the highest chemical shift near 177 ppm. The signals, referring to aromatic and azomethine carbon atoms, appeared between 106 and 163 ppm. In case of compounds **5a**–**e**, signals for morpholine ring carbon atoms appeared around 57.0 and 66.0 ppm. The ethyl chain between the morpholine and thioamide group showed peaks near 40.8 and 53.5 ppm, however the former carbon signals were overlapped by NMR solvent signal (DMSO-*d*_6_). Overall, proton integration and appropriate number of carbon signals in ^1^H and ^13^C NMR showed complete agreement with the corresponding structures. Furthermore, the elemental analyses of all the quinoline-thiosemicarbazones were in accordance with the proposed structures.

### 2.3. In Vitro Cholinesterase Inhibition and Structure–Activity Relationship Analyses

**Figure 2:**
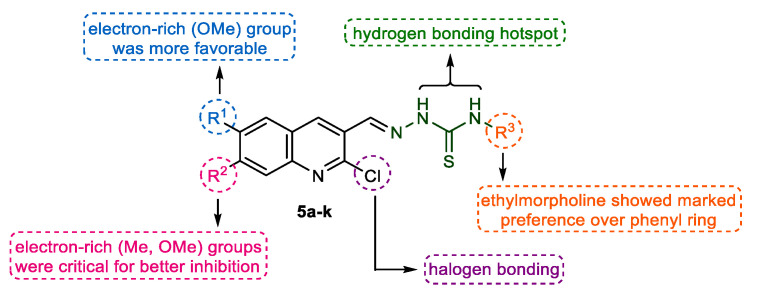
Representation of the diversity points and effect of chemical manipulation on biological activity.

The newly prepared quinoline-thiosemicarbazone hybrids **5a**–**k** were screened in vitro for their ability to inhibit cholinesterase enzymes (AChE and BChE) using Ellman’s method [56]. Galantamine was employed as a positive control. The results of inhibitory assays for target compounds are presented in Table 1. The designed molecules consist of two basic components: (i) the quinoline heterocycle with varied degree of structural features (R^1^ & R^2^), which is mainly involved in π-π stacking and π-alkyl interactions, and (ii) an acyclic thiosemicarbazone fragment bearing a suitable terminal attachment (R^3^) in the form of an aromatic ring (phenyl) and a saturated heterocyclic ring (morpholine) linked through an aliphatic linker (Figure 2). Various nitrogen atoms can act as hydrogen bond donor sites for establishing vital interactions with the amino acid residues in the active site of enzymes. Hence, the hybridization concept not only delivered new and diversified lead structures but also maintained significantly pharmacokinetically relevant parameters, such as molar mass. As such, various dynamic structure-activity relationship analyses could be manifested depicting the effect of functional group/substituent variation on the biological potential.

**Figure 3:**
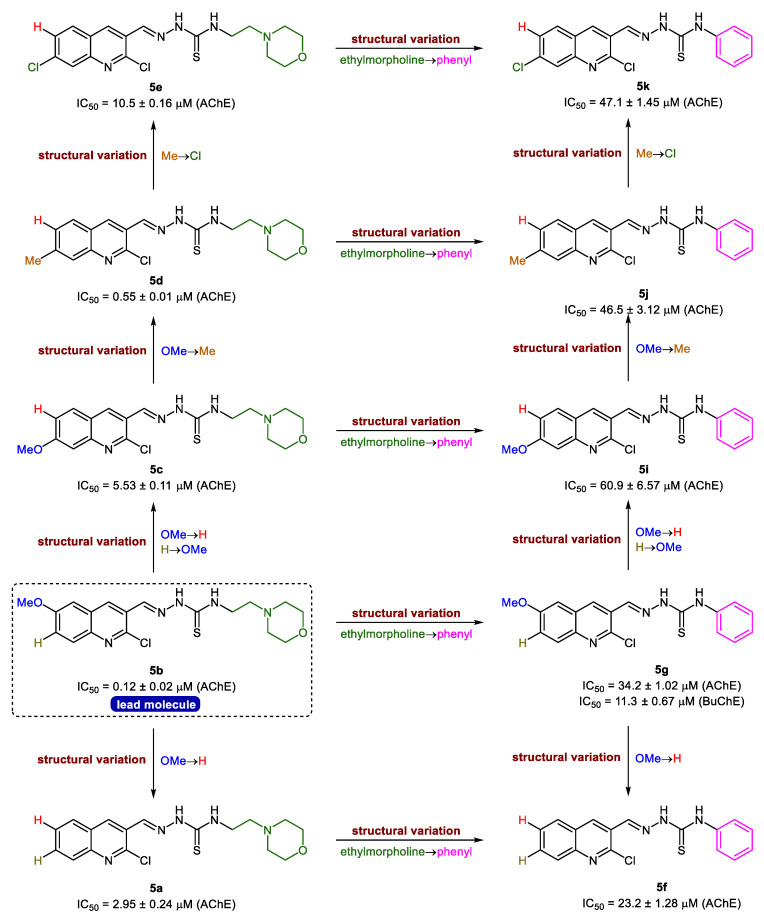
Structure-activity relationship analyses of quinoline-thiosemicarbazones **5a**–**k**.

The evaluation of activity results presented in Table 1 revealed that the intricate balance of substituents (R^1^, R^2^ & R^3^) is critical for the strong inhibitory effects. In general, all the tested hybrid derivatives were completely selective towards AChE and showed inhibition in the range of 0.12–60.9 μM. The presence of ethylmorpholine as R^3^ on the thioamide unit was significantly preferred over phenyl ring and the derivatives bearing this moiety were identified as the lead candidates. Compound **5b** showed the highest inhibitory efficacy with an IC_50_ value of 0.12 ± 0.02 μM, 5-fold more potent than the standard drug (galantamine; IC_50_ = 0.62 ± 0.01 μM). For further exploration of structure-activity relationships, various alterations were considered taking into account the substituents pattern on different sites of the hybrid structure (Figure 3). For instance, switching the position of methoxy group from 6-position (R^1^) to 7-position (R^2^) on quinoline ring led to reduced potency (**5c**; IC_50_ = 5.53 ± 0.11 μM), however, the effect of replacing ethylmorpholine with a phenyl ring was completely detrimental (**5i**; IC_50_ = 60.9 ± 6.57 μM). Replacement of methoxy group in **5c** with a methyl substituent reinstated the activity and compound **5d** showed slightly better inhibition profile than galantamine with an IC_50_ value of 0.55 ± 0.01 μM. Similar trend was observed for compound **5j**. Disappointingly, the introduction of a halogen substituent (Cl) as R^2^ produced deleterious effect in both cases (**5e** & **5k**); however, the compound bearing an ethylmorpholine (**5e**) was less affected compared to its congener (**5k**), which showed a sharp decrease in potency (IC_50_ = 47.1 ± 1.45 μM). Further modifications to compound **5b**, the lead molecule, were made while removing all the substituents at quinoline ring and the results unveiled the importance of methoxy substituent at 6-position for better in vitro inhibitory properties. The resulting compound **5a** showed significant activity (IC_50_ = 2.95 ± 0.24 μM), but less than lead inhibitor and standard drug (Figure 3). Collectively, the presence of both R^1^ and R^2^ substituents in combination with ethylmorpholine in an interactive manner is necessary for the quinoline-thiosemicarbazone hybrids to inhibit the acetylcholinesterase with maximum efficiency.

The synthesized hybrids **5a**–**k** have also been investigated for their potential to inhibit butyrylcholinesterase enzyme, however, the compounds showed exclusive selectivity towards acetylcholinesterase. Against butyrylcholinesterase, all the compounds showed <50% inhibition; however, in contrast to AChE results, the modest enhancement in potency preferred the presence of an aromatic (phenyl) ring instead of ethylmorpholine and a methoxy substituent appeared to be crucial for activity. The lead example was compound **5g** with an IC_50_ value of 11.3 ± 0.67 μM while other derivatives **5h**–**k** showed inhibition of BChE in the range of 16–32%. Hence, the results obtained for BChE confirmed the importance of aromatic moiety on the thioamide core, albeit low activity. 

### 2.4. Mechanism of Inhibition

With the help of kinetics studies, the mode of action of the most potent compounds **5b** and **5d** was determined using acetylthiocholine iodide as a substrate. Enzyme kinetics were used to determine the mechanism of acetylcholinesterase inhibition. Lineweaver-Burk graph (reciprocal of rate of reaction 1/S and reciprocal of substrate concentration 1/V) were used for the determination of the type of inhibition and analysis of effect of inhibitor on V_max_ and Kₘ. The slope K_m_/V_max_ of each line in the Lineweaver-Burk plot was plotted against different concentrations of substrate and chemicals to determine the value of K_i_. 

**Figure 4:**
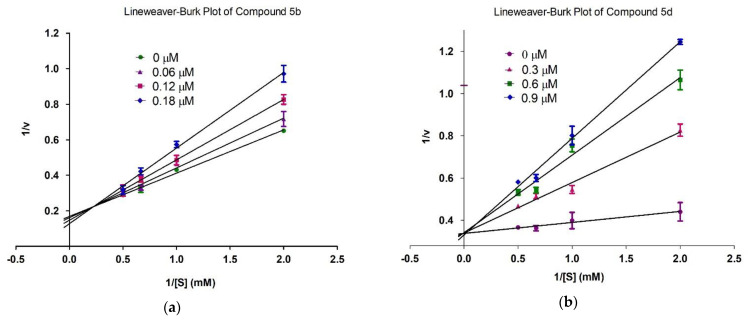
Inhibition of acetylcholinesterase by compounds **5b** (**a**) and **5d** (**b**). Lineweaver-Burk graph showing reciprocal rate of reaction 1/V against reciprocal of substrate 1/S.

Kinetics studies were performed on **5b** and **5d** with different concentrations of compounds and substrate. Four concentrations of 1 mM compound **5b** (0, 0.06, 0.12, 0.18 µM), compound **5d** (0, 0.3, 0.6, 0.9 µM) and four concentrations of substrate (0, 0.5, 1.0, 1.5 and 2.0 mM showing 1/S as 0.0, 2.0, 1.0, 0.667 and 0.5, respectively) were made. Both compounds competes with substrate (acetylthiocholine iodide) for binding in the active site of acetylcholinesterase. V_max_ of enzyme was not affected and K_m_ of acetylcholinesterase was increased which showed competitive inhibition as shown in Figure 4. In competitive inhibition, lines intersecting at y-axis show no change in V_max_ with an increase in the value of K_m_.

### 2.5. Molecular Docking Studies

**Figure 5:**
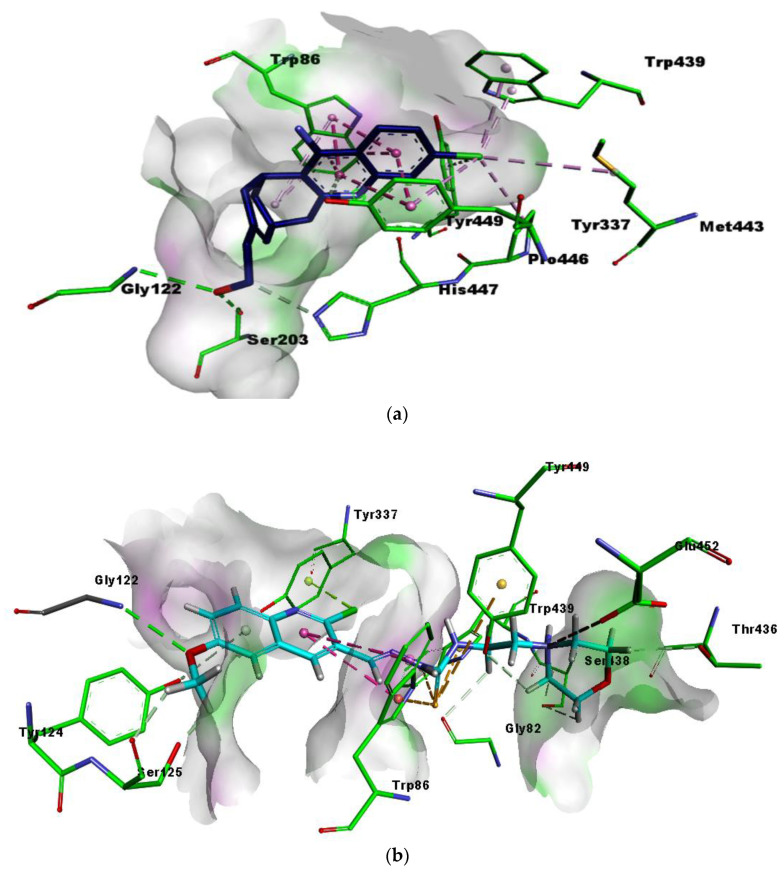

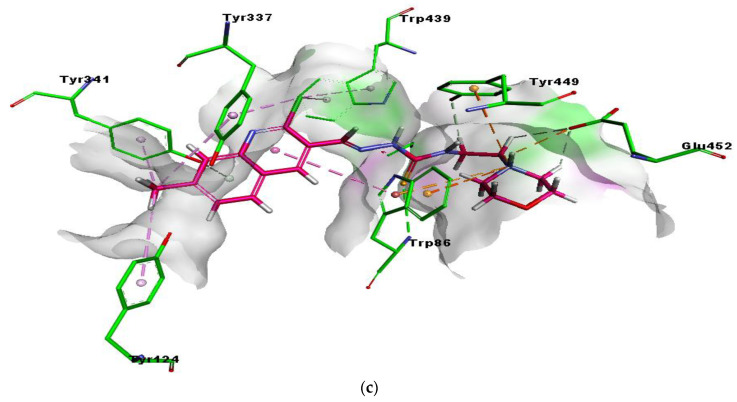
3D interactions of **huprine W** (**a**), compounds **5b** (**b**) and **5d** (**c**) with amino acid residues. π-π T-shaped interactions are shown as fuscia, hydrogen bond as green, π-sulfur as yellow and π-alkyl interaction as light green dashed lines.

For docking studies, X-ray structures of human AChE (PDB ID: 4BDT) [57] was selected due to the high crystallographic resolution (3.10 Å) of electric eel AChE. Molecular docking analysis of all the tested compounds was performed against AChE to explore the possible binding modes. An overview of the active site of AChE containing cognate ligand and all the inhibitors was presented in Figure S3. The orientation of the most potent and selective compounds **5b** and **5d** along with the crystallographic inhibitor **huprine W** were presented in the active site of AChE (Figure 5). 

The active pocket of AChE was surrounded by amino acid residues Leu76, Tyr124, Phe338, Gly122, Trp286, Tyr337, Val 340, Phe297, Leu289, Tyr72, Ser298, Ser125, Arg 296, Ser203, Tyr341, Ala204 and His447. The hydrogen bonds and π-π interactions were noticed by the most potent inhibitor **5b** as well as by **huprine W** as reported previously [45]. The cognate ligand (**huprine W**) showed two conventional hydrogen bonds with Ser203 (2.33 Å) and Gly122 (2.96 Å) and multiple π-π stacking interactions (4.00, 5.30, 4.41 and 3.69 Å) with Trp86. Additionally, 2-alkyl linkages (4.18 and 4.87 Å) with Trp439 and Tyr449, an alkyl linkage (4.52 Å) with Pro446 were also observed. Moreover, **huprine W** formed a π-π stacked bond (2.54 Å), one π-alkyl bond (4.45 Å) and π-donor hydrogen bond (4.01 Å) with Tyr337. Other interactions like π-alkyl with Met443 (4.89 Å) and a carbon-H bond (3.53 Å) with His447 were also present (Figure 5a). 

The most potent compound **5b** presented multiple important interactions with amino acids of active pocket like π-π T-shaped (6.23 Å) by quinoline ring and π-sulfur (4.34 Å) by sulfur atom in the thiosemicarbazone moiety with Trp86, the most important amino acid residue within the active pocket of AChE. Hydrogen bonding was noticed between oxygen atom and Gly122 (2.82 Å). However, Trp439 (4.68 Å) and Tyr449 (5.86 Å) exhibited π-sulfur interactions with sulfur atom in the thiosemicarbazone moiety of **5b**. Methoxy oxygen showed hydrogen bond with Gly122 (2.82 Å), while chloro atom formed π-lone pair (2.80 Å) with Tyr337. Moreover, nitrogen atom of morpholine ring exhibited attractive charge interactions (4.93 Å) with Glu452 (Figure 5b). It was clearly noted that methoxy group did not occupied the deep cleft of catalytic site. 

Another potent and selective compound **5d** docked inside the AChE represented several important interactions. These include π-π T-shaped (5.43 Å) by the methyl quinoline ring, hydrogen bond (3.30 Å), by the sulfur atom of thiosemicarbazone moiety and π-cation interactions (5.26 Å), and by the nitrogen atom of morpholine ring with Trp86. Additionally, the nitrogen atom of morpholine ring was involved in the formation of the salt bridge (2.69 Å) andπ-cation (4.78 Å), and interactions with Glu452 and Tyr449, respectively. Moreover, multiple π-alkyl (5.14, 5.37 and 3.76 Å) interactions were formed by methyl group with Tyr337, Tyr124 and Tyr341, respectively. The chloro group at the quinoline ring showed π-alkyl interactions (5.43 and 3.32 Å) with Trp439 and Tyr337, respectively, as shown in Figure 5c. Both compounds exhibited mostly similar interactions with the same residues, except for the presence of 6-methoxy and 7-methyl at the quinoline ring. These multiple interactions, especially π-π and strong hydrogen bonds by the potent compounds deep inside the catalytic cleft of acetylcholinesterase, can be the possible reason for their significant inhibitory profile. 

The binding poses of all the derivatives were shown in Figure S3, which clearly represents the binding of compound **5b** and **5d** near cognate ligand showing interactions with important residues as **Huprine W** does. However, other residues do not show binding at the same position, instead showed binding with other amino acids and are slightly away from the active pocket

The type of interactions between ligands (**5b** and **5d**) and receptor (4BDT) along with the distance and atoms involved in the interactions are presented in 2D interactions in Figure S4 and Table 2. The docking studies revealed that the most potent compound **5b**, having 6-methoxy at quinoline ring was responsible for the conformational changes and the best binding of compound within the active pocket of enzyme. In vitro results of all the compounds showing variable inhibition against acetylcholinesterase were justified from their binding interactions within the catalytic region of the enzyme. Moreover, the selected compounds presented negative free energy values and were found to bind with significant affinity. Taken together, results presented herein showed that quinoline morpholinoethyl hydrazine carbothioamide derivatives are promising inhibitors of acetylcholinesterase.

### 2.6. HYDE Assessment of Potent Inhibitors against Acetylcholinesterase

The top 20 docked conformations were assessed using the HYDE affinity method [58]. The assessment was made for the selected ligands using LeadIT software. The docking scores, binding free energies, and the most favorable poses for all the derivatives were calculated using FlexX (Table 3). The results demonstrated that the potent inhibitors (**5b** and **5d**) have high affinity towards the active site of acetylcholinesterase, as depicted by binding free energies. The potent inhibitors bind to the receptor with a very high binding affinity compared to the cognate ligands of the enzyme. Moreover, moderately and less active compounds exhibited low binding affinity as compared to potent compounds. 

### 2.7. SeeSAR Visual Drug Design

**Figure 6:**
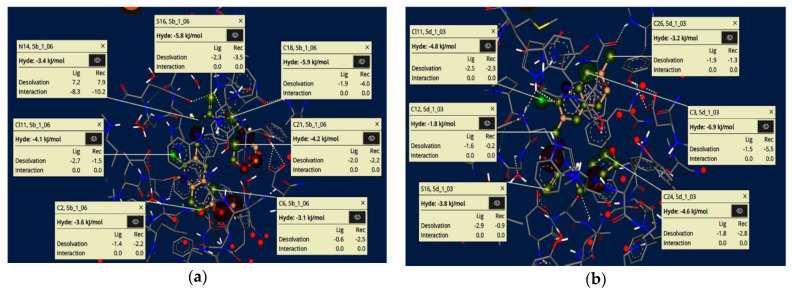
Visual and investigative modes of the docked pose of compounds **5b** (**a**) and **5d** (**b**) within the active site of acetylcholinesterase (pdb: 4BDT). The green colour represents the favourable and contributing atoms showing interactions inside the active pocket, while light green are chloride atoms, and yellow are sulfur atoms.

The visual and investigative modes of the docked pose of compounds **5b** and **5d** revealed interpretable, innovative and important conformations using the SeeSAR tool in the LeadIT software [58,59]. Figure 6 represents the iterative and interactive optimization of leads showing the binding and non-binding capacity of compounds. Desolvation and interactions for compounds **5b** and **5d** are also shown in Figure 6. The approach predicts the visual and interpretable feedback for implicit hydrogen bonds and dehydration, and confirms our molecular docking results obtained using FlexX default parameters.

### 2.8. Molecular Dynamics Simulations

In addition to the molecular docking studies, we have performed molecular simulations for the cognate ligand (**Hup W**) and the most potent compound **5b** showing many fold higher inhibitory activity compared to other inhibitors. The MD simulations of the enzyme in complex with the cognate ligand (**Hup W**) and selected inhibitor **5b** were carried out in an aqueous environment for 30 ns with initial conformations taken from docking pose having the lowest binding free energy. Noncovalent interactions between ligands, **Hup W**, and **5b** and the active site of acetylcholinesterase were monitored in a time dependent manner.

**Figure 7:**
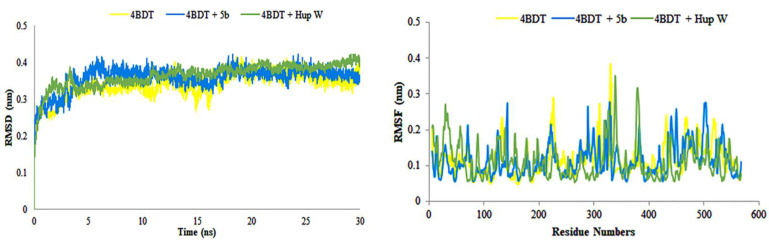
Root mean square deviations (RMSD) and root mean square fluctuations (RMSF) of amino acid residues of protein (4BDT) during 30 ns simulation time in the absence and presence of compound **5b**.

The results of MD simulations are shown in the form of RMSD values, which give information regarding the overall stability of protein and its complex with the inhibitor. As shown in Figure 7, cognate ligand (**Hup W**) exhibited stability from the very start soon after 5 ns and was stable for rest of the time course. The only deviation found was at 2–4.8 ns. The selected ligand (**5b**) showed significant stability and less deviation within the range of 0.3–0.4 nm. The little variations were noticed in the structure of apo protein, whereas the structure of complex was found significantly stable after 4 ns. The structure of **5b**+protein complex showed little deviation between 14–17 ns, while remaining simulations were found stable as compared to protein alone. The consistent slight fluctuations were noted for protein during the whole simulation time (Figure 7). 

The knowledge of root mean square fluctuation values give the information about the calculations that were carried out to check the flexibility of the structure of receptor in the absence and presence of compound. As shown in Figure 7, the systems having apo and holo proteins presented noteworthy pattern of fluctuations. The apo protein started from 0.2 nm and showed the fluctuation between 0.1 and 0.3 nm during the simulation time with slight increase to 0.4 nm, whereas the holo protein, (protein + **5b**) started from 0.15 nm with fluctuation between 0.1 and 0.28 nm during the simulation time with slight increase. However, the holo protein, (protein + **Hup W**) showed fluctuations within the range of 0.10 to 0.30 during the simulation time course (30 ns). Overall, the system showed stability and less fluctuation. The region having motifs and loops showed less fluctuations, while active site pocket acquired significant stability during the whole time course of simulations. The results recommended the overall stability of complex as compared to protein alone. The results of protein structure depicted the stability of internal motion in protein and complex systems.

**Figure 8:**
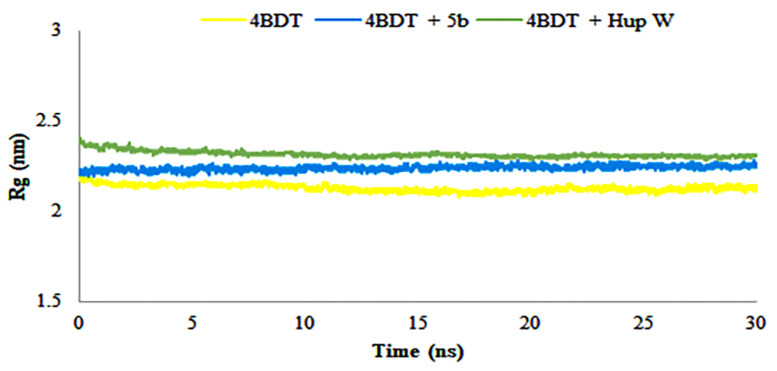
Radius of gyration (Rg) of amino acid residues in the presence and absence of compound **5b** during 30 ns MD simulation run.

The radius of gyration was calculated to determine the compactness of the system during MD simulation time. It also describes the folding and unfolding of protein structure in the absence and presence of cognate ligand (**Hup W**) and compound **5b**. The results provided in Figure 8, demonstrated the compactness of system alone and in the presence of selected cognate ligand and compound. The average scores of Rg for acetylcholinesterase and its complex with **Hup W** and compound **5b** were found to be 2.3 and 2.25 nm, respectively, and showed the compactness of structures throughout the simulations. The results contribute towards the stability and compactness of protein only, with **Hup W** and compound **5b** during the simulation time, therefore playing a significant role in the increased affinity of compound **5b** for acetylcholinesterase enzyme.

### 2.9. ADME Properties

Pharmacokinetic properties of compounds **5b** and **5d** were predicted to assess the impact of different parameters using previously reported prediction tools [60,61,62,63]. These parameters include molecular weight, polar surface area, number of atom types (donor/acceptor), molecular refractivity, and lipophilicity (i.e., the partition coefficient, such as log Po/w, n-octanol, WLOGP, MLOGP and XLOGP3, etc.) representing the free energies of solvation and solvent accessible surface area [64]. Moreover, water solubility predicts solubility of the compounds. These properties predict the drug-likeness and blood-brain barrier permeation of test compounds. The results presented in Table 4 suggested that the tested derivatives are safe to use as drugs. However, in case of BBB permeation, the compounds showed poor pharmacokinetics and may not be able to cross BBB, while all the other properties exhibited by selected compounds are favorable.

## 3. Materials and Methods

### 3.1. General

The chemicals and solvents used were of analytical grade and obtained from commercial suppliers Scharlau (Barcelona, Spain), Merck (Darmstadt, Germany), and Fluka (Buchs, Switzerland), and were used without further purification. Thin layer chromatography was performed using aluminum plates coated with silica gel 60F_254_ (Merck) in an appropriate eluent. The spots were visualized using ultraviolet irradiation. Melting points were recorded on Gallenkamp melting point apparatus (UK) and were uncorrected. ^1^H NMR spectra were recorded in DMSO-d_6_ solvent on a Bruker Avance NMR (300 MHz, Billerica, MA, USA) spectrometer while ^13^C NMR spectra were recorded at 75 MHz. Chemical shifts are reported as δ values in parts per million (ppm) relative to tetramethylsilane as internal standard. Coupling constant (*J*) is given in Hertz. FTIR spectra were recorded on an Agilent Technologies Cary 630 FTIR (Santa Clara, CA, USA). Elemental analysis was performed on a LECO 630-200-200 TRUSPEC CHNS micro analyzer (St. Joseph, MI, USA) and the values observed were within ± 0.4% of the calculated results.

Acetylcholinesterase (E.C.3.1.1.7, from electric eel), butyrylcholinesterase (E.C. 3.1.1.8, from horse serum), substrates acetythiocholine chloride, butyrylthiocholine chloride and 5,5-dithio-bis-[2-nitrobenzoic acid] were purchased from Sigma-Aldrich (St. Louis, MO, USA) and Merck (Darmstadt, Germany). Galantamine was used as a standard drug. A 96-well microplate reader (BioTek ELx800, Instruments, Inc. Winooski, VT, USA) was used to determine the biological activities of the compounds.

### 3.2. Preparation of Acetanilides ***2a**–**f***

Orthophosphoric acid (0.2 mol) was added to a stirred solution of substituted anilines **1a**–**f** (1.0 mol) in glacial acetic acid (2.0 mol, 118 mL) and the resulting mixture was heated to reflux for 6 h. After the completion of the reaction, the mixture was poured into ice cold water with continuous stirring. The precipitated solid was filtered off, washed with excess cold water, and recrystallized from boiling water to produce acetanilides **2a**–**f** [45].

### 3.3. Preparation of 2-Chloroquinoline-3-carbaldehydes ***3a**–**f***

A Vilsmeier reagent, prepared from dropwise addition of POCl_3_ (65.3 mL, 0.70 mol) to DMF (19.3 mL, 0.25 mol) at 0 °C with continuous stirring, was added corresponding acetanilide **2a**–**f** (0.10 mol). The resulting mixture was heated to 80 °C for 6–18 h. After the completion of the reaction (monitored by TLC), the reaction mixture was cautiously poured onto crushed ice (500 g) and stirred for 30 minutes at 0–10 °C. The precipitated solid was filtered off, washed with excess water, dried, and recrystallized from ethyl acetate to produce 2-chloroquinoline-3-carbaldehydes **3a**–**f** [55].

### 3.4. General Procedure for the Preparation of Quinoline-Thiosemicarbazones ***5a**–**k***

To a stirred solution of *N*-(2-morpholinoethyl)hydrazinecarbothioamide **4a** or *N*-phenylhydrazinecarbothioamide **4b** (1 mmol) in absolute ethanol (20 mL) was added 2-chloroquinoline-3-carbaldehyde **3** (1 mmol) followed by orthophosphoric acid (10 mol%). The resulting reaction mixture was heated to reflux for 2 h. The precipitated solid was filtered off and recrystallized from methanol to afford quinoline-thiosemicarbazones **5a**–**k**.

*2-((2-Chloroquinolin-3-yl)methylene)-N-(2-morpholinoethyl)hydrazinecarbothioamide***5a:** Yield 92%. Yellow solid. Mp 250–252 °C. FTIR (cm^−1^): 3340 (N-H), 3149 (N-H), 2995 (C-H), 1654 (C=N), 1112 (C=S); ^1^H NMR (DMSO-*d*_6_, 300 MHz) δ_H_ = 2.46 (t, *J* = 4.5 Hz, 4H, NCH_2_ of morpholine ring), 2.55 (t, *J* = 6.6 Hz, 2H, NH-CH_2_-CH_2_-morpholine), 3.61 (t, *J* = 4.5 Hz, 4H, OCH_2_ of morpholine ring), 3.70 (q, *J* = 6.6 Hz, 2H, NH-CH_2_-CH_2_-morpholine), 7.23 (td, *J* = 7.5, 0.9 Hz, 1H, ArH), 7.33 (d, *J* = 8.1 Hz, 1H, ArH), 7.53 (td, *J* = 7.5, 1.2 Hz, 1H, ArH), 7.65 (dd, *J* = 7.8, 0.9 Hz, 1H, ArH), 8.30 (s, 1H, N=CH), 8.54–8.57 (m, 2H, NH, ArH), 11.76 (s, 1H, =N-NH); ^13^C NMR (DMSO-*d*_6_, 75 MHz) δ_C_ = 40.8, 53.7, 57.0, 66.8, 115.7, 119.5, 123.0, 125.9, 128.8, 131.5, 134.9, 137.1, 139.4, 161.4, 177.4; Anal. Calcd. for C_17_H_20_ClN_5_OS: C, 54.03; H, 5.33; N, 18.53; S, 8.49%. Found: C, 53.85; H, 5.01; N, 18.33; S, 8.20%.

*2-((2-Chloro-6-methoxyquinolin-3-yl)methylene)-N-(2-morpholinoethyl)hydrazinecarbothioamide***5b:** Yield 89%. Off-white solid. Mp 226–228 °C. FTIR (cm^−1^): 3120 (N-H), 3003 (C-H), 1617 (C=N), 1110 (C=S); ^1^H NMR (DMSO-*d*_6_, 300 MHz) δ_H_ = 2.47 (t, *J* = 4.5 Hz, 4H, NCH_2_ of morpholine ring), 2.57 (t, *J* = 6.9 Hz, 2H, NH-CH_2_-CH_2_-morpholine), 3.62 (t, *J* = 4.5 Hz, 4H, OCH_2_ of morpholine ring), 3.73 (q, *J* = 6.3 Hz, 2H, NH-CH_2_-CH_2_-morpholine), 3.90 (s, 3H, ArOCH_3_), 7.27 (d, *J* = 2.7 Hz, 1H, ArH), 7.46 (dd, *J* = 9.3, 2.7 Hz, 1H, ArH), 7.85 (d, *J* = 9.3 Hz, 1H, ArH), 8.48 (s, 1H, N=CH), 8.66 (t, *J* = 5.7 Hz, 1H, NH), 8.93 (s, 1H, ArH), 11.91 (s, 1H, =N-NH); ^13^C NMR (DMSO-*d*_6_, 75 MHz) δ_C_ = 40.9, 53.7, 56.1, 56.9, 66.8, 106.1, 124.6, 126.7, 128.4, 129.8, 134.5, 137.2, 143.5, 146.3, 158.5, 177.6; Anal. Calcd. for C_18_H_22_ClN_5_O_2_S: C, 53.00; H, 5.44; N, 17.17; S, 7.86%. Found: C, 53.25; H, 5.70; N, 17.34; S, 8.01%.

*2-((2-Chloro-7-methoxyquinolin-3-yl)methylene)-N-(2-morpholinoethyl)hydrazinecarbothioamide***5c:** Yield 90%. Yellow solid. Mp 242–244 °C. FTIR (cm^−1^): 3291 (N-H), 3156 (N-H), 3001 (C-H), 1667 (C=N), 1107 (C=S); ^1^H NMR (DMSO-*d*_6_, 300 MHz) δ_H_ = 2.46 (t, *J* = 4.5 Hz, 4H, NCH_2_ of morpholine ring), 2.56–2.60 (m, 2H, NH-CH_2_-CH_2_-morpholine), 3.60–3.72 (m, 6H, OCH_2_ of morpholine ring, NH-CH_2_-CH_2_-morpholine), 3.94 (s, 3H, ArOCH_3_), 6.82 (d, *J* = 1.8 Hz, 1H, ArH), 6.87 (dd, *J* = 8.7, 2.4 Hz, 1H, ArH), 7.55 (d, *J* = 9.0 Hz, 1H, ArH), 8.26 (s, 1H, N=CH), 8.63 (t, *J* = 5.4 Hz, 1H, NH), 8.97 (s, 1H, ArH), 11.94 (s, 1H, =N-NH); ^13^C NMR (DMSO-*d*_6_, 75 MHz) δ_C_ = 41.0, 53.7, 55.9, 57.0, 66.8, 107.0, 113.8, 122.6, 124.1, 130.4, 134.9, 137.6, 141.4, 149.5, 162.4, 177.2; Anal. Calcd. for C_18_H_22_ClN_5_O_2_S: C, 53.00; H, 5.44; N, 17.17; S, 7.86%. Found: C, 52.92; H, 5.30; N, 17.05; S, 7.77%.

*2-((2-Chloro-7-methylquinolin-3-yl)methylene)-N-(2-morpholinoethyl)hydrazinecarbothioamide***5d:** Yield 87%. Yellow solid. Mp 256–258 °C. FTIR (cm^−1^): 3332 (N-H), 3197 (N-H), 2851 (C-H), 1655 (C=N), 1106 (C=S); ^1^H NMR (DMSO-*d*_6_, 300 MHz) δ_H_ = 2.46–2.53 (m, 9H, NCH_2_ of morpholine ring, NH-CH_2_-CH_2_-morpholine, ArCH_3_), 3.60 (t, *J* = 4.2 Hz, 4H, OCH_2_ of morpholine ring), 3.71 (q, *J* = 6.6 Hz, 2H, NH-CH_2_-CH_2_-morpholine), 7.54 (d, *J* = 8.4 Hz, 1H, ArH), 7.74 (s, 1H, ArH), 7.89 (d, *J* = 8.1 Hz, 1H, ArH), 8.49 (s, 1H, N=CH), 8.66 (t, *J* = 5.4 Hz, 1H, NH), 9.00 (s, 1H, ArH), 11.89 (s, 1H, =N-NH); ^13^C NMR (DMSO-*d*_6_, 75 MHz) δ_C_ = 22.0, 41.0, 53.7, 57.0, 66.7, 125.3, 125.8, 127.2, 128.4, 130.7, 135.7, 137.4, 142.6, 147.7, 161.5, 177.5; Anal. Calcd. for C_18_H_22_ClN_5_OS: C, 55.16; H, 5.66; N, 17.87; S, 8.18%. Found: C, 55.40; H, 5.84; N, 17.98; S, 8.30%.

*2-((2,7-Dichloroquinolin-3-yl)methylene)-N-(2-morpholinoethyl)hydrazinecarbothioamide***5e:** Yield 91%. Pale Yellow solid. Mp 237–239 °C. FTIR (cm^−1^): 3330 (N-H), 3479 (N-H), 3001 (C-H), 1602 (C=N), 1102 (C=S); ^1^H NMR (DMSO-*d*_6_, 300 MHz) δ_H_ = 2.46 (t, *J* = 3.0 Hz, 4H, NCH_2_ of morpholine ring), 2.56–2.59 (m, 2H, NH-CH_2_-CH_2_-morpholine), 3.58–3.61 (m, 4H, OCH_2_ of morpholine ring), 3.73 (q, *J* = 6.4 Hz, 2H, NH-CH_2_-CH_2_-morpholine), 7.76 (dd, *J* = 8.7, 2.1 Hz, 1H, ArH), 8.03–8.09 (m, 2H, ArH), 8.51 (s, 1H, N=CH), 8.70 (t, *J* = 5.7 Hz, 1H, NH), 9.12 (s, 1H, ArH), 11.95 (s, 1H, =N-NH); ^13^C NMR (DMSO-*d*_6_, 75 MHz) δ_C_ = 41.1, 53.7, 57.0, 66.8, 126.0, 127.2, 129.3, 130.7, 132.7, 136.6, 140.1, 147.5, 150.2, 153.7, 177.6; Anal. Calcd. for C_17_H_19_Cl_2_N_5_OS: C, 49.52; H, 4.64; N, 16.98; S, 7.78%. Found: C, 49.80; H, 4.88; N, 17.12; S, 7.96%.

*2-((2-Chloroquinolin-3-yl)methylene)-N-phenylhydrazinecarbothioamide***5f:** Yield 94%. Yellow solid. Mp 251–253 °C. FTIR (cm^−1^): 3303 (N-H), 3157 (N-H), 3046 (C-H), 1653 (C=N), 1193 (C=S); ^1^H NMR (DMSO-*d*_6_, 300 MHz) δ_H_ = 7.20–7.27 (m, 2H, ArH), 7.32–7.43 (m, 3H, ArH), 7.51–7.60 (m, 3H, ArH), 7.68 (dd, *J* = 8.1, 0.9 Hz, 1H, ArH), 8.41 (s, 1H, N=CH), 8.85 (s, 1H, ArH), 10.18 (s, 1H, NH), 12.06 (s, 1H, =N-NH); ^13^C NMR (DMSO-*d*_6_, 75 MHz) δ_C_ = 115.7, 119.6, 122.9, 125.6, 126.0, 126.6, 128.6, 129.0, 131.6, 135.9, 137.9, 139.5, 161.5, 176.5; Anal. Calcd. for C_17_H_13_ClN_4_S: C, 59.91; H, 3.84; N, 16.44; S, 9.41%. Found: C, 60.09; H, 4.00; N, 16.72; S, 9.65%.

*2-((2-Chloro-6-methoxyquinolin-3-yl)methylene)-N-phenylhydrazinecarbothioamide***5g:** Yield 88%. Light brown solid. Mp 225–227 °C. FTIR (cm^−1^): 3368 (N-H), 3295 (N-H), 3031 (C-H), 1656 (C=N), 1157 (C=S); ^1^H NMR (DMSO-*d*_6_, 300 MHz) δ_H_ = 3.90 (s, 3H, ArOCH_3_), 7.26 (tt, *J* = 7.2, 1.2 Hz, 1H, ArH), 7.32 (d, *J* = 3.0 Hz, 1H, ArH), 7.39–7.50 (m, 3H, ArH), 7.56–7.60 (m, 2H, ArH), 7.87 (d, *J* = 9.3 Hz, 1H, ArH), 8.61 (s, 1H, N=CH), 9.27 (s, 1H, ArH), 10.28 (s, 1H, NH), 12.21 (s, 1H, =N-NH); ^13^C NMR (DMSO-*d*_6_, 75 MHz) δ_C_ = 56.1, 106.3, 124.6, 126.2, 126.5, 126.8, 128.6, 128.7, 129.7, 135.4, 138.1, 139.4, 143.6, 146.5, 158.5, 176.9; Anal. Calcd. for C_18_H_15_ClN_4_OS: C, 58.30; H, 4.08; N, 15.11; S, 8.65%. Found: C, 58.58; H, 4.24; N, 15.33; S, 8.91%.

*2-((2-Chloro-6-methylquinolin-3-yl)methylene)-N-phenylhydrazinecarbothioamide***5h:** Yield 92%. Light brown solid. Mp 242–244 °C. FTIR (cm^−1^): 3303 (N-H), 3151 (N-H), 2995 (C-H), 1651 (C=N), 1123 (C=S); ^1^H NMR (DMSO-*d*_6_, 300 MHz) δ_H_ = 2.34 (s, 3H, ArCH_3_), 7.22–7.28 (m, 3H, ArH), 7.60 (d, *J* = 8.4 Hz, 2H, ArH), 7.68 (dd, *J* = 8.4, 1.8 Hz, 1H, ArH), 7.77 (s, 1H, ArH), 7.86 (d, *J* = 8.7 Hz, 1H, ArH), 8.40 (s, 1H, N=CH), 8.81 (s, 1H, ArH), 10.15 (s, 1H, NH), 12.19 (s, 1H, =N-NH); ^13^C NMR (DMSO-*d*_6_, 75 MHz) δ_C_ = 21.6, 126.5, 126.7, 127.4, 128.0, 128.7, 134.3, 136.0, 137.6, 138.0, 139.4, 146.1, 148.2, 161.3, 176.8; Anal. Calcd. for C_18_H_15_ClN_4_S: C, 60.92; H, 4.26; N, 15.79; S, 9.04%. Found: C, 61.14; H, 4.48; N, 15.95; S, 9.18%.

*2-((2-Chloro-7-methoxyquinolin-3-yl)methylene)-N-phenylhydrazinecarbothioamide***5i:** Yield 91%. Yellow solid. Mp 224–226 °C. FTIR (cm^−1^): 3294 (NH), 3149 (NH), 2931 (CH), 1609 (C=O), 1121 (C=S); ^1^H NMR (DMSO-*d*_6_, 300 MHz) δ_H_ = 3.94 (s, 3H, ArOCH_3_), 6.83 (dd, *J* = 6.0, 2.4 Hz, 1H, ArH), 6.87 (d, *J* = 2.4 Hz, 1H, ArH), 7.22–7.43 (m, 5H, ArH), 7.91 (d, *J* = 9.0 Hz, 1H, ArH), 8.61 (s, 1H, N=CH), 9.28 (s, 1H, ArH), 10.25 (s, 1H, NH), 12.14 (s, 1H, =N-NH); ^13^C NMR (DMSO-*d*_6_, 75 MHz) δ_C_ = 56.0, 107.1, 113.9, 122.2, 123.9, 125.9, 126.5, 128.6, 130.6, 136.1, 138.4, 139.5, 141.5, 149.6, 162.3, 176.3; Anal. Calcd. for C_18_H_15_ClN_4_OS: C, 58.30; H, 4.08; N, 15.11; S, 8.65%. Found: C, 58.44; H, 4.20; N, 15.30; S, 8.80%.

*2-((2-Chloro-7-methylquinolin-3-yl)methylene)-N-phenylhydrazinecarbothioamide***5j:** Yield 90%. Yellow solid. Mp 205–207 °C. FTIR (cm^−1^): 3301 (N-H), 3143 (N-H), 2936 (C-H), 1653 (C=N), 1197 (C=S); ^1^H NMR (DMSO-*d*_6_, 300 MHz) δ_H_ = 2.53 (s, 3H, ArCH_3_), 7.37–7.44 (m, 3H), 7.53 (dd, *J* = 8.7, 1.5 Hz, 1H, ArH), 7.59 (d, *J* = 7.5 Hz, 2H, ArH), 7.75 (s, 1H, ArH), 7.91 (d, *J* = 8.4 Hz, 1H, ArH), 8.61 (s, 1H, N=CH), 9.31 (s, 1H, ArH), 10.28 (s, 1H, NH), 12.18 (s, 1H, =N-NH); ^13^C NMR (DMSO-*d*_6_, 75 MHz) δ_C_ = 22.0, 117.5, 124.5, 125.6, 126.2, 126.8, 127.2, 128.7, 130.6, 136.4, 138.2, 139.4, 142.7, 147.8, 161.6, 176.8; Anal. Calcd. for C_18_H_15_ClN_4_S: C, 60.92; H, 4.26; N, 15.79; S, 9.04%. Found: C, 60.78; H, 4.10; N, 15.57; S, 8.88%.

*2-((2,7-Dichloroquinolin-3-yl)methylene)-N-phenylhydrazinecarbothioamide***5k:** Yield 93%. Yellow solid. Mp 237–239 °C. FTIR (cm^−1^): 3270 (N-H), 3090 (C-H), 1655 (C=N), 1197 (C=S); ^1^H NMR (DMSO-*d*_6_, 300 MHz) δ_H_ = 7.36–7.45 (m, 3H, ArH), 7.57–7.59 (m, 3H, ArH), 7.72 (dd, *J* = 8.7, 2.1 Hz, 1H, ArH), 8.04 (s, 1H, ArH), 8.60 (s, 1H, N=CH), 9.39 (s, 1H, ArH), 10.29 (s, 1H, NH), 12.23 (s, 1H, =N-NH); ^13^C NMR (DMSO-*d*_6_, 75 MHz) δ_C_ = 126.0, 126.6, 126.8, 127.1, 128.7, 129.1, 130.8, 135.8, 136.6, 137.6, 139.3, 147.6, 150.3, 161.3, 176.9; Anal. Calcd. for C_17_H_12_Cl_2_N_4_S: C, 54.41; H, 3.22; N, 14.93; S, 8.54%. Found: C, 54.63; H, 3.38; N, 15.07; S, 8.70%.

### 3.5. In Vitro Cholinesterase Inhibition Assay

Inhibition of acetylcholinesterase and butyrylcholinesterase was measured in vitro by spectrophotometric method developed by Ellman [56] with slight modifications [65]. Briefly, the reaction mixture contained 60 μL phosphate buffers (KH_2_PO_4_/KOH), pH 7.7, 10 μL of test compound dissolved in DMSO (final DMSO concentration was 2%) and 10 μL of enzyme (0.5 and 3.4 U/mg of AChE or BChE, respectively). Reaction contents were mixed thoroughly and kept for 10 min during pre-incubation at 37 °C. After the pre-incubation, 10 μL of 1 mM acetythiocholine chloride or butyrylthiocholine chloride was added to the respective AChE or BChE enzyme solution to start the enzymatic reactions. DTNB (10 µL, 0.5 mM) was also added as a coloring reagent. The reaction mixture was again incubated for 20 min at 37 °C and the absorbance was measured at 405 nm using 96-well micro-plate reader. All experiments were carried out in triplicate. Galantamine (0.1 mM) was used as a standard inhibitor. In order to measure the activity of enzyme, assay was performed with a blank containing all of the components except inhibitor. The percent inhibition was calculated by the following formula:

Inhibition (%) = 100 − (A_c_/A_f_) × 100 (1)

Where “A_c_” and “A_f_” are absorbance obtained for the respective enzyme (AChE and BChE) in the presence or absence of inhibitors, after subtracting the respective background (pre-read absorbance). Compounds exhibiting > 50% inhibition against ChEs were further evaluated for the determination of IC_50_ values which were calculated by non-linear curve fitting program PRISM 5.0 (GraphPad, San Diego, CA, USA).

### 3.6. Kinetics Studies

Michaelis-Menten kinetics experiments were used to determine the type of enzyme inhibition. Detailed kinetics studies of the potent compounds **5b** and **5d** were performed to probe the potential mechanism of action to inhibit the enzyme. For this purpose, the initial rates of the enzyme inhibition at four concentrations of substrate (0, 0.5, 1.0, 1.5, 2.0 mM) in the absence and presence of four different concentrations of compound **5b** (0, 0.06, 0.12, 0.18 µM) and compound **5d** (0, 0.3, 0.6, 0.9 µM) against acetylcholinesterase were measured.

### 3.7. Molecular Docking Protocols

#### 3.7.1. Structure Selection and Preparation

Molecular docking studies were conducted to investigate the putative interactions of the compounds making complex with the acetylcholinesterase enzyme. In order to perform docking studies, the crystallographic structure of human AChE (PDB ID: 4BDT) was obtained from the RCSB PDB database [57], and prepared for the docking analysis. Prior to the experiments, the structures of the enzyme and compounds were prepared as follows. The enzyme structure was protonated with the Protonate3D [66] algorithm implemented within the molecular modeling tool MOE [67]. The structure was energy minimized using Amber99 force field including all crystallographic solvent molecules. The backbone atoms were restrained with a small force in order to avoid collapse of the binding pockets during energy minimization calculations. Subsequently, the co-crystallized ligands and solvent molecules were removed. The crystallographic water molecules were removed and hypothetical hydrogen atoms were added to the X-ray structure in standard geometries with the MOE.

#### 3.7.2. Compounds Preparation

The 3D structural coordinates of compounds were generated using MOE followed by assignment of protonation and ionization states in physiological pH range by using the “wash” module. Afterwards, the structures of compounds were energy minimized with the MMFF94x force field for docking studies.

#### 3.7.3. Docking Studies

For docking studies, calculations were performed using LeadIT from BioSolveIT, GmbH Germany [68]. Receptor was loaded by Load or Prepare Receptor utility of the LeadIT software. The binding site for the receptor was defined in 9.0 Å spacing of the amino acid residues. By FlexX utility of LeadIT, docking of compounds was performed. For this purpose, compounds were docked inside the active site of receptor and 50 conformations for each ligand-receptor complex were produced based on binding free energies. Default docking parameters were not modified and top 30 highest scoring docked positions were kept for further analysis [58]. Poses with lowest free binding energy values were considered as the most stable ones having the highest affinity to interact with the receptor. Each ligand-protein complex having lowest binding free energy for interactions was examined and 3D putative binding modes were visualized using Discovery Studio Visualizer v4 [69].

### 3.8. Molecular Dynamics Simulations

The crystallographic structure of human AChE (PDB ID: 4BDT) [57] was obtained from the Protein Data Bank (www.pdb.org, accessed on 14 September 2021). Protein manipulation and protonation were made with the help of GROMOS96 force field having the 43a1 parameter set. The GROMACS (Groningen Machine for Chemical Simulation) simulation packages, 5.1.4 were used for the MD simulations and protocol for molecular dynamics simulations was used according to previously developed methods [70,71,72] with little modifications. Parameterization of compound 5b and Huprine W were done online using the PRODRG servers [73]. MOE and VMD [74] were used for the visualization and molecular inspection. The crystallographic structure was solvated (addition of water molecule) and counter ions were incorporated to neutralize the receptor. Subsequently, the energy minimization of the system was done, followed by equilibration using two sequential NVT (100 ps) and NPT (100 ps) runs during which protein’s heavy atoms were restrained.

After minimization, the resulting ensembles were submitted to 30 ns MD simulations with a time-step of 2 fs for each simulation. Periodic boundary conditions (PBC) were applied during all the simulations. Steepest descent method was used for simple energy minimizations. All NVT and NPT runs used the Berendsen thermostat and the Parrinello-Rahman barostat for temperature (approx. 303 K) and pressure coupling (approx. 1.01 bar), respectively. The cut-off radius of 10 Å and smooth Particle Mesh Ewald (PME) protocol were observed for long-range method. The root mean square deviations, fluctuations and radius of gyration were plotted using XMGRACE v5.1.19 [75].

## 4. Conclusions

In summary, a series of new quinoline-thiosemicarbazone hybrids was designed and synthesized using a facile multistep protocol. Several commercially available anilines were successfully employed to construct quinoline heterocycle via Vilsmeyer-Haack formylation reaction. Hybridization of quinoline carbaldehydes with thiosemicarbazides afforded target compounds in excellent yields, devoiding the need of column chromatographic purification. Evaluation of cholinesterase inhibitory potential revealed the discovery of numerous potent and highly efficacious inhibitors. In particular, compound **5b** inhibited the acetylcholinesterase selectively showing an IC_50_ value of 0.12 ± 0.02 μM, a 5-fold high potency than galantamine (standard). Structure-activity relationship analysis showed the importance of electron-rich (methoxy) substituent at quinoline ring and ethylmorpholine on the carbothioamide unit, playing a vital role in obtaining high therapeutic efficacy. Docking, physicochemical properties, lipophilicity, water solubility, pharmacokinetics, drug-likeness, and medicinal chemistry properties were also calculated for the potent inhibitors suggesting the safer profile to be investigated as drug molecules and have high probability of blood-brain penetration and absorption. Collectively, our findings established that compound **5b** is a potent, selective and competitive inhibitor of acetylcholinesterase and can serve as a promising candidate for further preclinical development for the therapy of Alzheimer’s disease. 

## Figures and Tables

**Table 1:** Anti-cholinesterase (AChE and BChE) potential of synthesized compounds **5a**–**k**.

Compound	Substituent			Acetylcholinesterase(AChE)	Butyrylcholinesterse(BChE)
	R^1^	R^2^	R^3^	IC_50_ ± SEM (μM)/%inhibition	
**5a**	H	H	ethylmorpholine	2.95 ± 0.24	3.32%
**5b**	OMe	H	ethylmorpholine	0.12 ± 0.02	8.12%
**5c**	H	OMe	ethylmorpholine	5.53 ± 0.11	1.33%
**5d**	H	Me	ethylmorpholine	0.55 ± 0.01	1.52%
**5e**	H	Cl	ethylmorpholine	10.5 ± 0.16	1.88%
**5f**	H	H	Ph	23.2 ± 1.28	4.47%
**5g**	OMe	H	Ph	34.2 ± 1.02	11.3 ± 0.67
**5h**	Me	H	Ph	49.3 ± 2.49	32.6%
**5i**	H	OMe	Ph	60.9 ± 6.57	24.0%
**5j**	H	Me	Ph	46.5 ± 3.12	16.8%
**5k**	H	Cl	Ph	47.1 ± 1.45	20.3%
**Galantamine**	―	―	―	0.62 ± 0.01	0.87 ± 0.03

**Table 2:** The types of binding interactions, distance of bonds and atoms involved in interactions.

Compounds	Binding interactions			
	Ligand Atom	Receptor Atom	Interaction Type	Distance (Å)
**5b**	O26	GLY122	H-bond	2.82
	phenyl ring	TRP86	π-π T-shaped & π-sulfur	6.23 & 4.34
	Cl11	TYR337	π-lone pair	2.80
	S16	TRP439	π-sulfur	4.68
	N20	GLU452	Attractive charges	4.93
	S16	TYR449	π-sulfur	5.86
**5d**	N20	GLU452	salt bridge	2.69
	N20	TYR449	π-cation	4.78
	S16, phenyl ring & N20	TRP86	H-bond, π-π T-shaped & π-cation	3.30, 5.43 & 5.26
	Cl11	TRP439	π-alkyl	5.43
	Methyl & Cl11	TYR337	π-alkyl	5.14 & 3.32
	Methyl	TYR124	π-alkyl	5.37
	Methyl	TYR341	π-alkyl	3.76
**Hup W**	O1	GLY122	H-bond	2.96
	O1	SER203	H-bond	2.33
	Quinoline ring	TRP86	π-π Stacked & π-alkyl	4.41, 5.30, 4.00, 3.69 & 4.18, 4.87
	Cl1	PRO446	Alkyl	4.52
	Cl1	TYR337	π-π Stacked & π-alkyl, π-donor	4.47, 2.54 & 4.45, 4.01
	Cl1	MET443	Alkyl	4.89
	Cl1	TRP439	π-alkyl	3.46 & 3.80
	Cl1	TYR449	π-alkyl	5.38

**Table 3:** Docking scores, binding free energies and their corresponding ranks by Hyde affinity assessment.

Compound	Docking Score by FlexX	Pose Rank	Binding Free Energy ΔG (kJ/mol)
**5a**	−22.12	1	−17
**5b**	−26.58	2	−25
**5c**	−21.56	2	−16
**5d**	−23.01	3	−18
**5e**	−19.67	2	−16
**5f**	−14.55	4	−12
**5g**	−14.62	3	−10
**5h**	−13.69	1	−8
**5i**	−12.58	2	−9
**5j**	−14.24	1	−10
**5k**	−13.99	2	−9
**Huprine W**	−16.29	1	−23

**Table 4:** ADMET prediction scores for the selected compounds.

Properties.	Compounds	
	5b	5d
**Physicochemical Properties**		
Molecular weight (g/mol)	407.92	391.92
No. of heavy atoms	27	26
No. of aromatic heavy atoms	10	10
Fraction C_sp3_	0.39	0.39
No. of rotatable bonds	8	7
No. of H-bond acceptors	5	4
No. of H-bond donors	2	2
Molar refractivity	114.72	113.20
TPSA (Å^2^)	103.10	93.87
**Lipophilicity**		
Log *P*_o/w_ (iLOGP)	3.65	3.21
Log *P*_o/w_ (XLOGP3)	2.47	2.86
Log *P*_o/w_ (WLOGP)	1.65	1.95
Log *P*_o/w_ (MLOGP)	0.97	1.50
Log *P*_o/w_ (SILICOS-IT)	3.96	4.41
Consensus Log *P*_o/w_	2.54	2.79
**Water Solubility**		
Log S (ESOL)	−3.67	−3.89
Solubility (mg/mL; mol/L)	8.70 × 10^−2^; 2.13 × 10^−4^	5.00 × 10^−2^; 1.28 × 10^−4^
Class	Soluble	Soluble
Log S (ALi)	−4.28	−4.49
Solubility(mg/ml; mol/l)	2.14 × 10^−2^; 5.26 × 10^−5^	1.27 × 10^−2^; 3.23 × 10^−5^
Class	Moderately soluble	Moderately soluble
Log S (SILICOS-IT)	−5.70	−5.98
Solubility (mg/ml; mol/l)	8.06 × 10^−4^; 1.98 × 10^−6^	4.13 × 10^−4^; 1.05 × 10^−6^
Class	Moderately soluble	Moderately soluble
**Pharmacokinetics**		
GI absorption	High	High
BBB permeant	No	No
P-gp substrate	Yes	Yes
CYP1A2 inhibitor	Yes	Yes
CYP2C19 inhibitor	Yes	Yes
CYP2C9 inhibitor	Yes	Yes
CYP2D6 inhibitor	No	Yes
CYP3A4 inhibitor	Yes	Yes
Log *K*_p_ (skin permeation) (cm/s)	−7.03	−6.66
**Druglikeness**		
Lipinski	Yes; 0 violation	Yes; 0 violation
Ghose	Yes; 0 violation	Yes; 0 violation
Veber	Yes; 0 violation	Yes; 0 violation
Egan	Yes; 0 violation	Yes; 0 violation
Muegge	Yes; 0 violation	Yes; 0 violation
Bioavailability score	0.55	0.55
**Medicinal Chemistry**		
PAINS	0 alert	0 alert
Brenk	3 alerts: 2-halo_pyridine, imine_1, thiocarbonyl_group	3 alerts: 2-halo_pyridine, imine_1, thiocarbonyl_group
Leadlikeness	No; 2 Violations: MW > 350, Rotors > 7	No; 1 Violation MW > 350
Synthetic accessibility	3.16	3.18

## Data Availability

The data presented in this study are available in Supplementary Material.

## Supplementary Materials

^1^H and ^13^C NMR spectra of all the synthesized compounds, dose response curves for enzyme (AChE and BChE) inhibition activity, comparative docking assessment results, 2D interactions of **huprine W**, compounds **5b** and **5d** with amino acid residues and Visual and investigative modes of the docked pose of compounds **5b** and **5d** within the active site of AChE are available online.

## Sample Availability

Samples of the synthetic compounds are available from the authors on reasonable request.
