# Supplementary material for: Hybrid Quinoline-Thiosemicarbazone Therapeutics as a New Treatment Opportunity for Alzheimer’s Disease‒Synthesis, In Vitro Cholinesterase Inhibitory Potential and Computational Modeling Analysis
Source: Molecules. 2021 Oct 30;26(21):6573. doi: 10.3390/molecules26216573 (PMC8587653; doi:10.3390/molecules26216573)
Supplement: Supplementary file 1 [file molecules-26-06573-s001.zip › molecules-1402448-supplementary.pdf]

Supporting Information

Hybrid Quinoline-Thiosemicarbazone Therapeutics as a New Treatment Opportunity for Alzheimer's Disease – Synthesis, In Vitro Cholinesterase Inhibitory Potential and Computational Modeling Analysis

Sumera Zaib [1,*], Rubina Munir [2,*], Muhammad Tayyab Younas [1], Naghmana Kausar [3], Aliya Ibrar [4], Sehar Aqsa [2], Noorma Shahid [2], Tahira Tasneem Asif [2], Hashem O. Alsaab [5] and Imtiaz Khan [6,*]

[1]*Department of Biochemistry, Faculty of Life Sciences, University of Central Punjab, Lahore 54590, Pakistan; muhammadtayyabyounassst@gmail.com (M.T.Y.)*

[2]*Department of Chemistry, Kinnaird College for Women, Lahore 54000, Pakistan; w14bche002@gmail.com (S.A.); tahiratasneem1998@gmail.com (T.T.A.)*

[3]*Department of Chemistry, University of Gujrat, Gujrat 50700, Pakistan; naghmana.kousar@uog.edu.pk (N.K.)*

[4]*Department of Chemistry, Faculty of Natural Sciences, The University of Haripur, Haripur, KPK 22620, Pakistan; aliya.ibrar@uoh.edu.pk (A.I.)*

[5]*Department of Pharmaceutics and Pharmaceutical Technology, Taif University, P.O. Box 11099, Taif 21944, Saudi Arabia; h.alsaab@tu.edu.sa (H.O.A.)*

[6]*Department of Chemistry and Manchester Institute of Biotechnology, The University of Manchester, 131 Princess Street, Manchester M1 7DN, UK*

*__Correspondence:__ sumera.zaib@ucp.edu.pk (S.Z.); rubina.munir@kinnaird.edu.pk (R.M.); imtiaz.khan@manchester.ac.uk (I.K.)

Supporting Information

NMR spectra of quinoline-thiosemicarbazones 5(a-k)

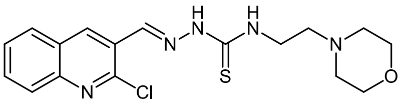

5a

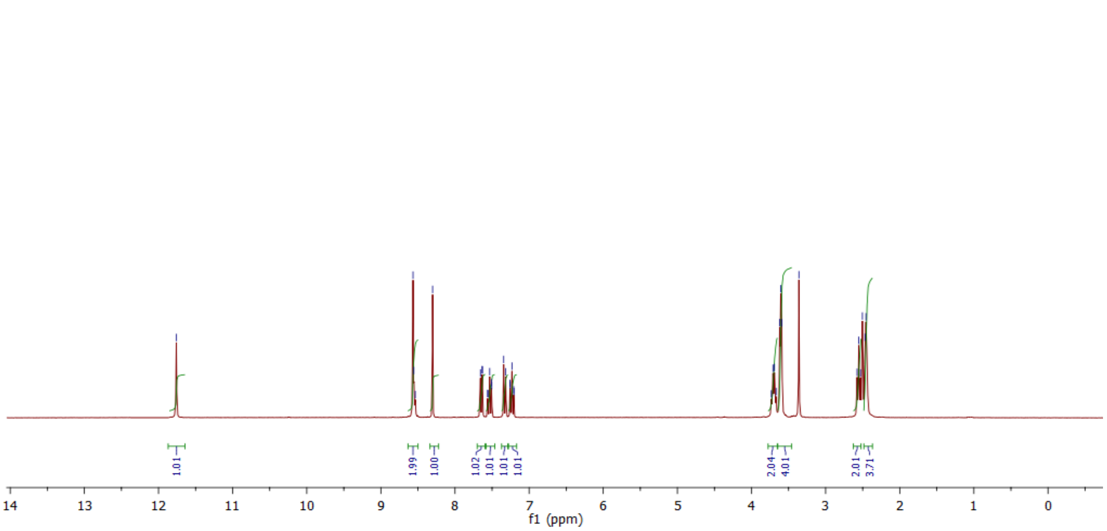

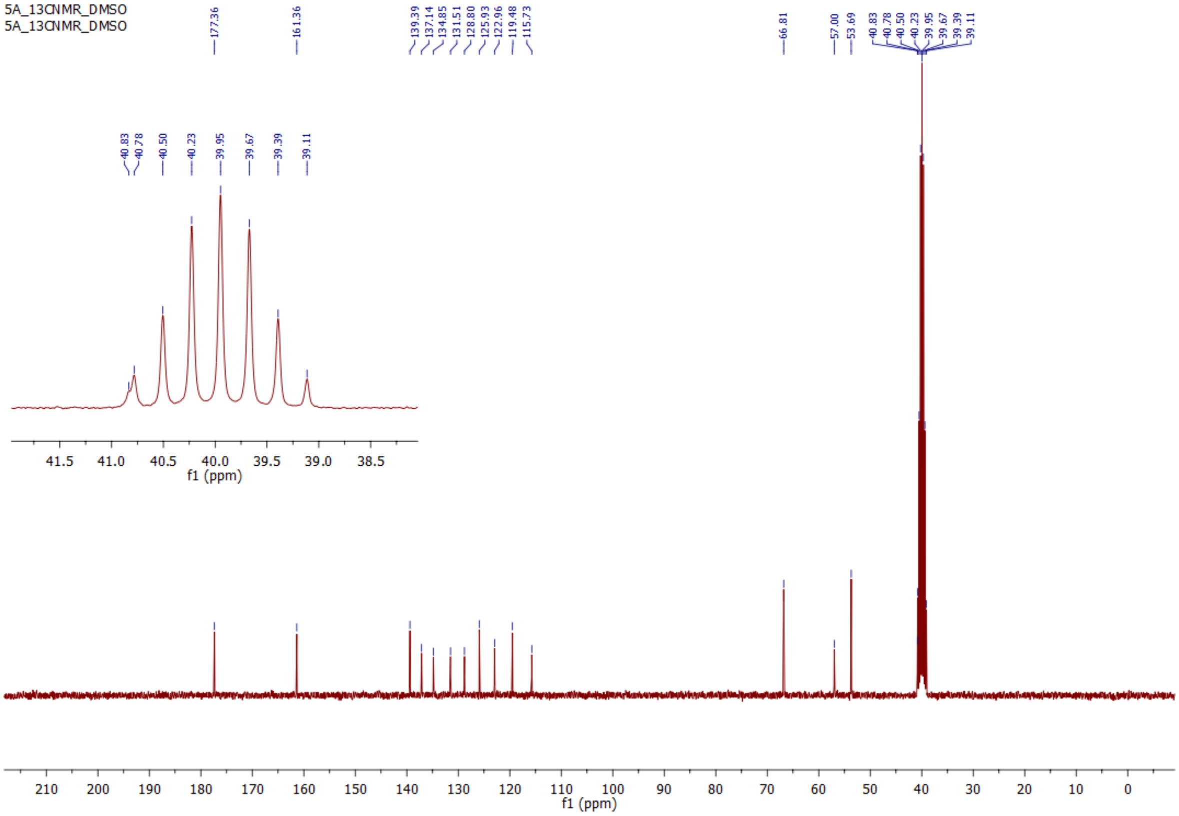

Supporting Information

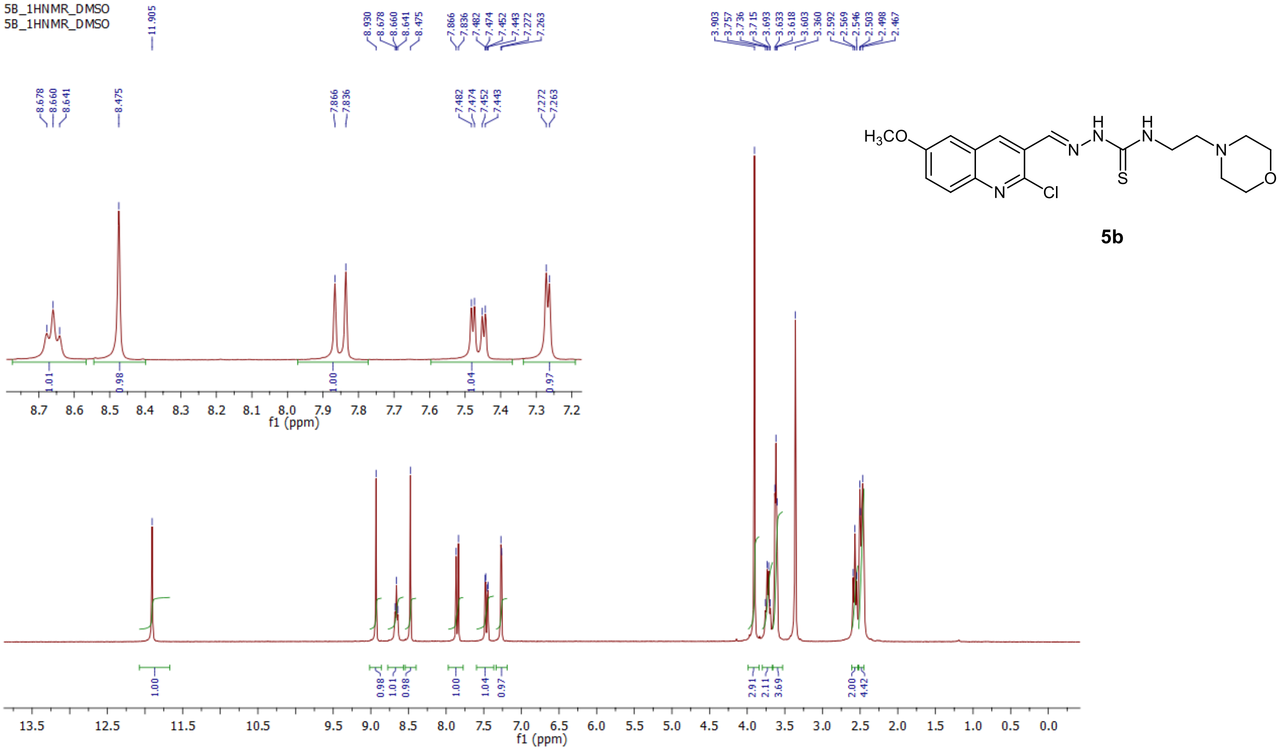

5b

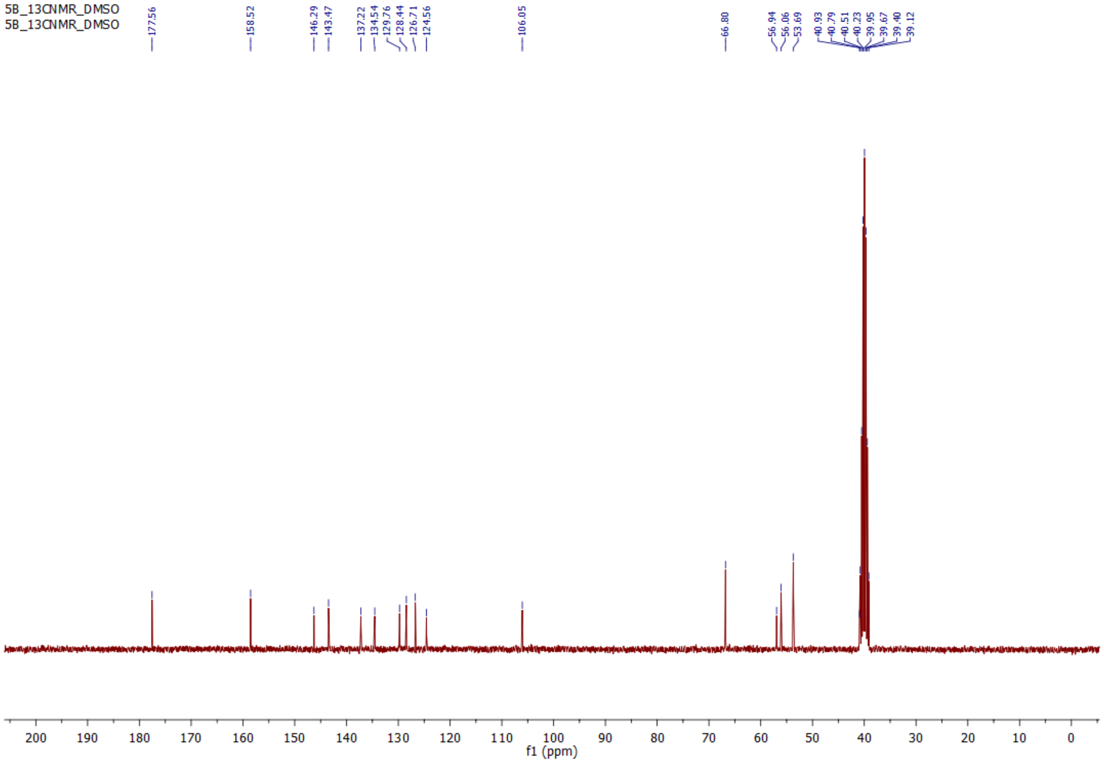

Supporting Information

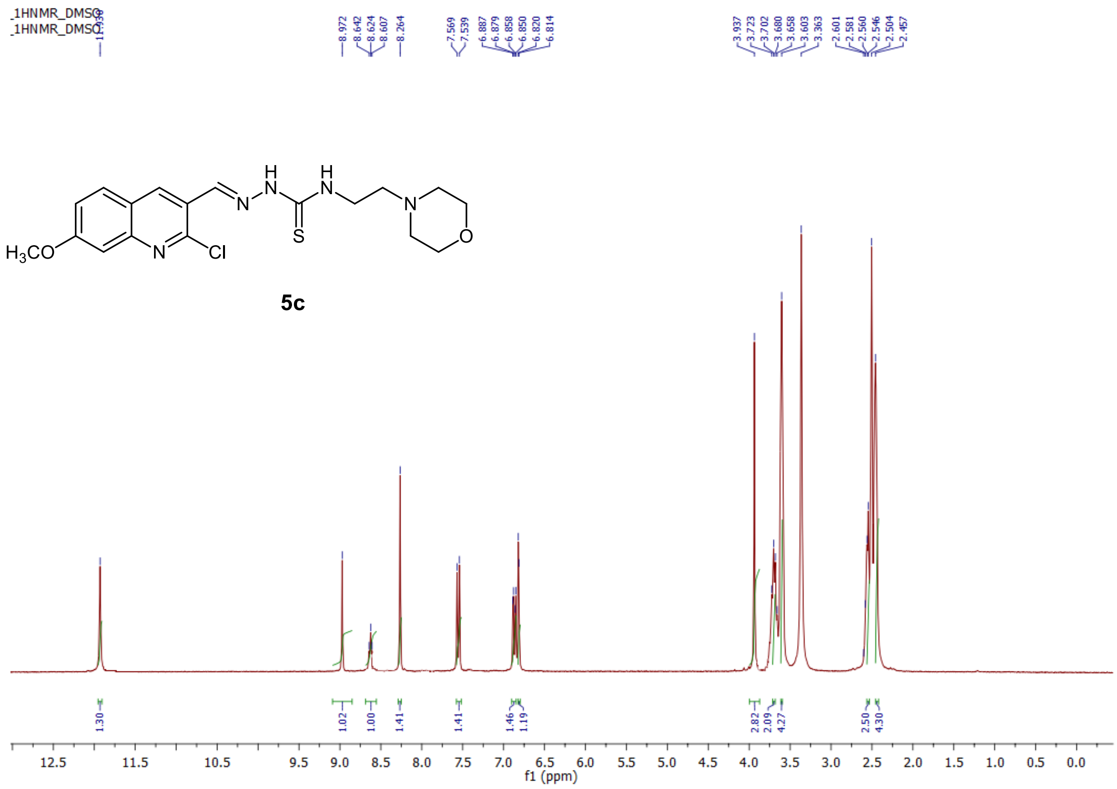

5c

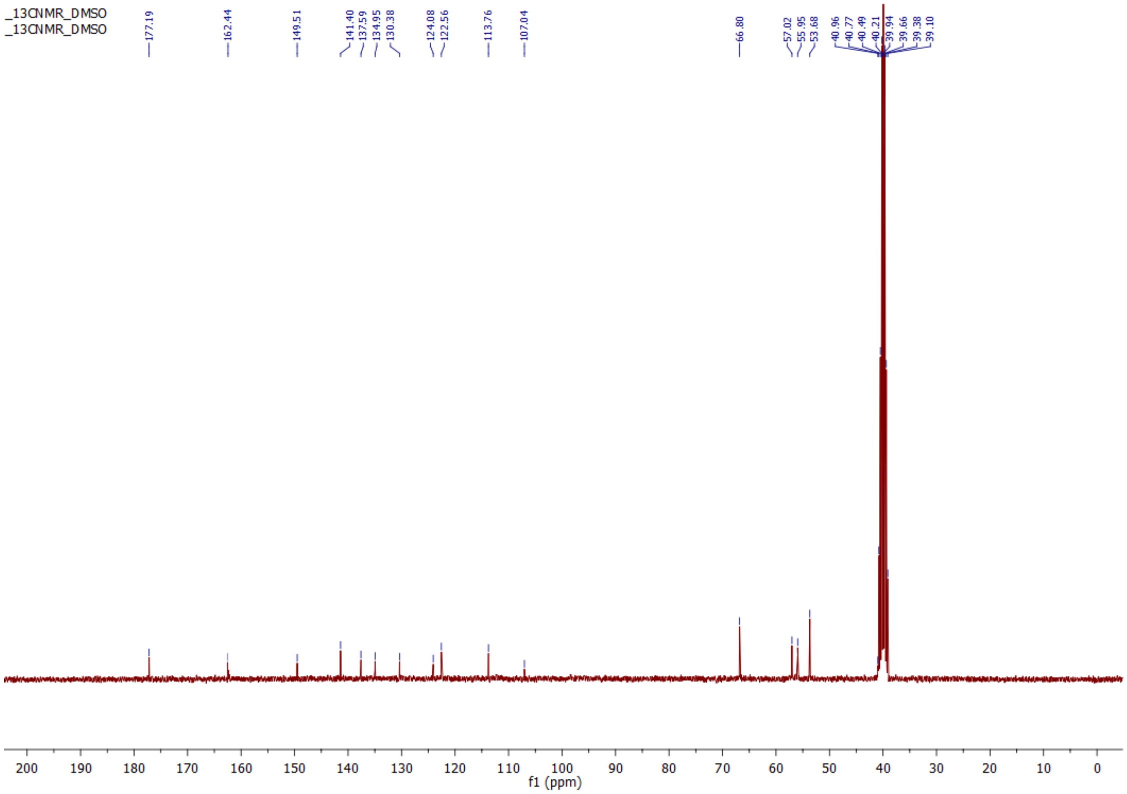

Supporting Information

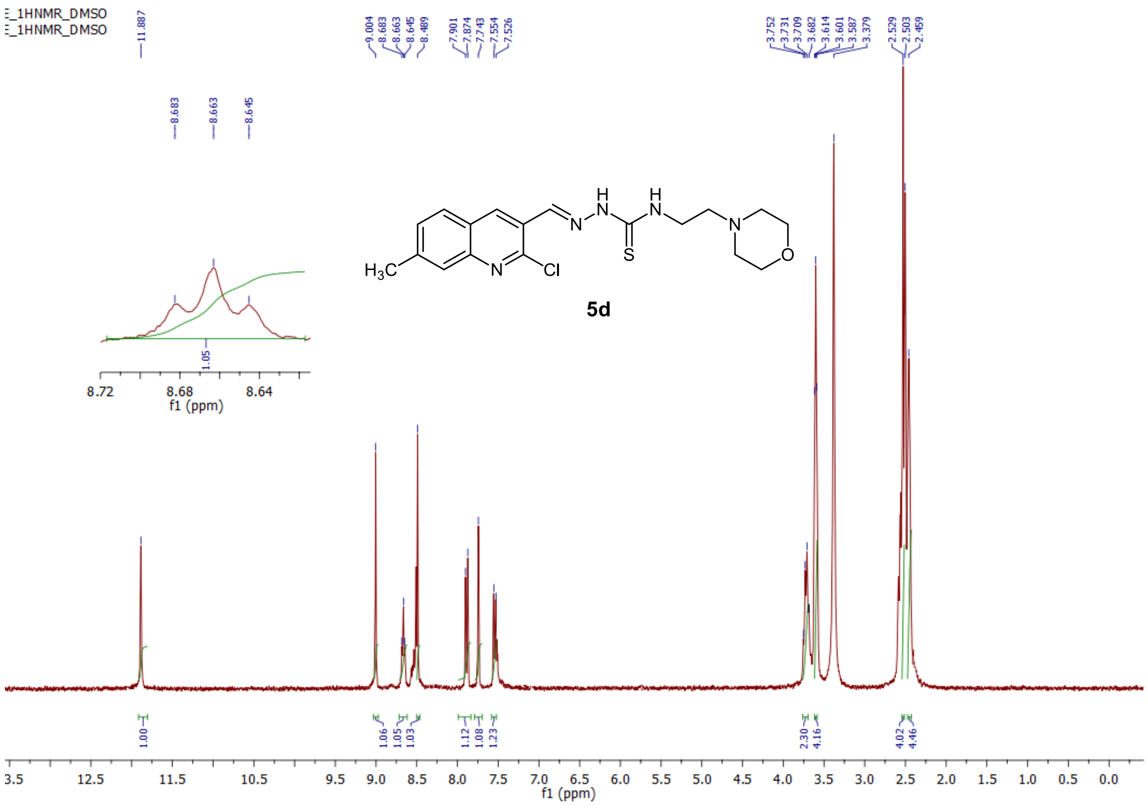

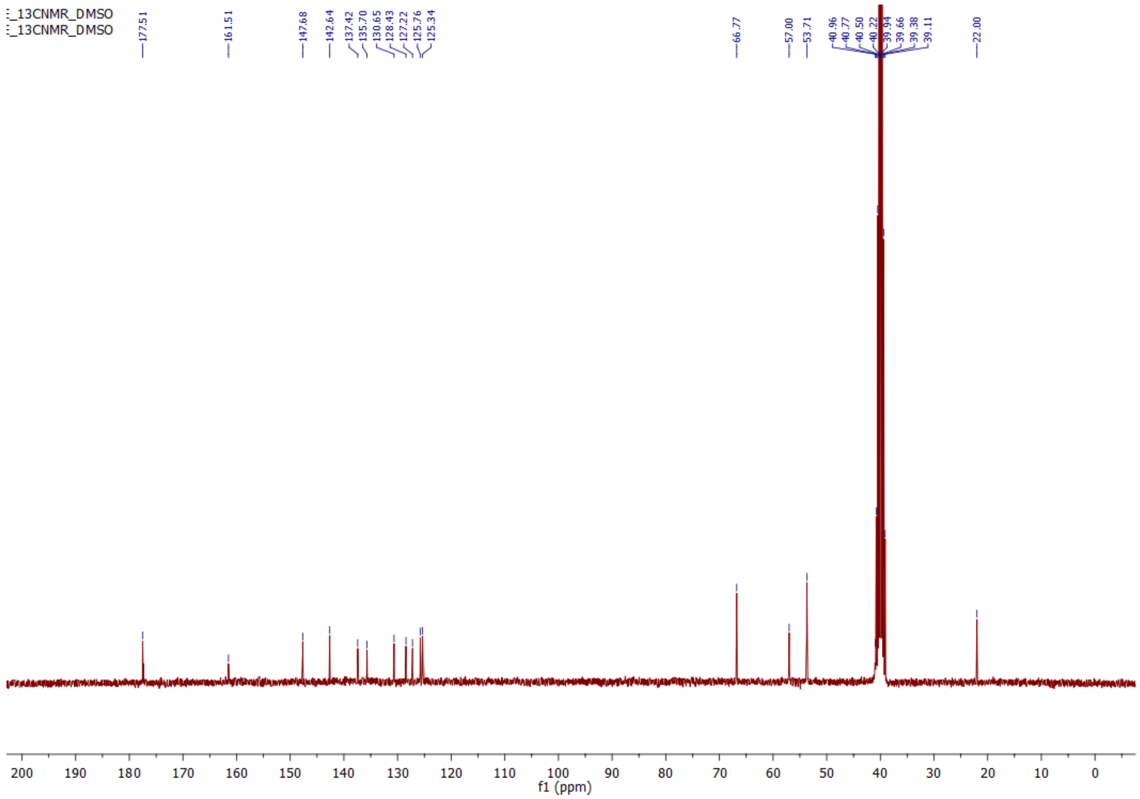

Supporting Information

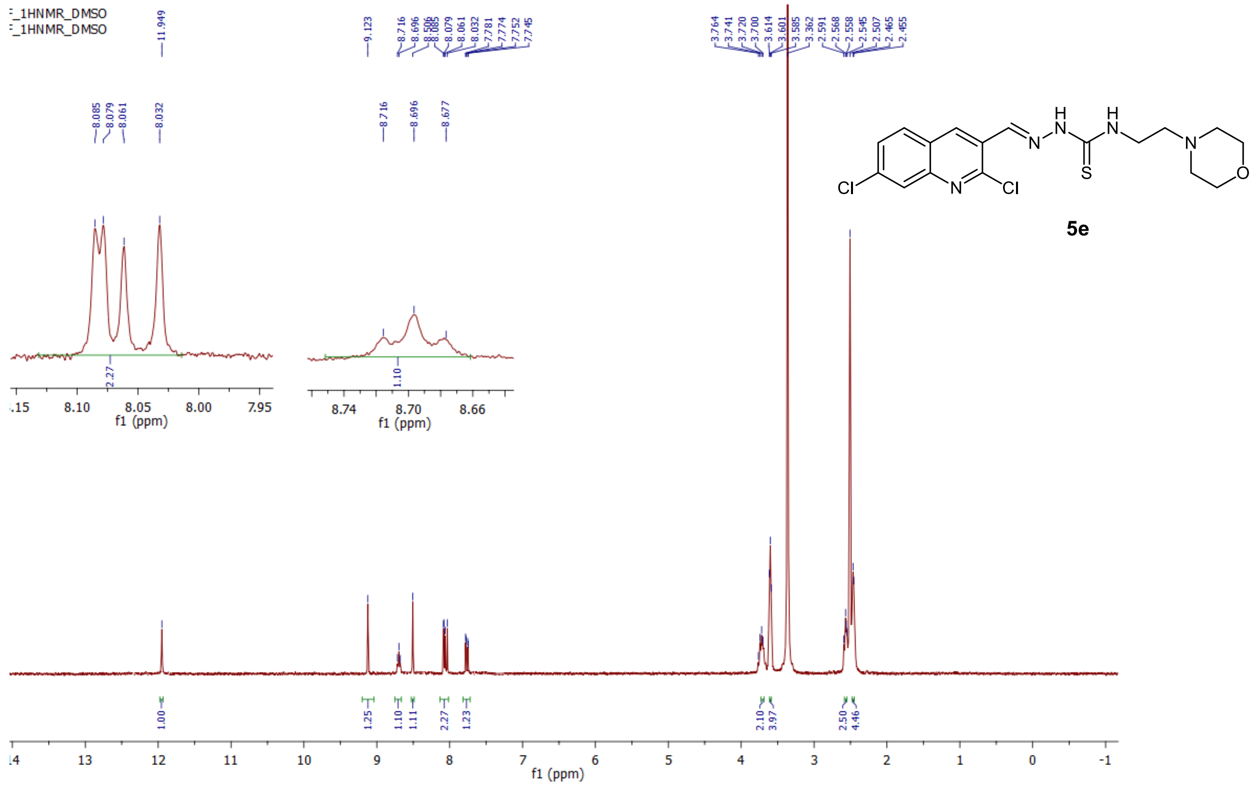

5e

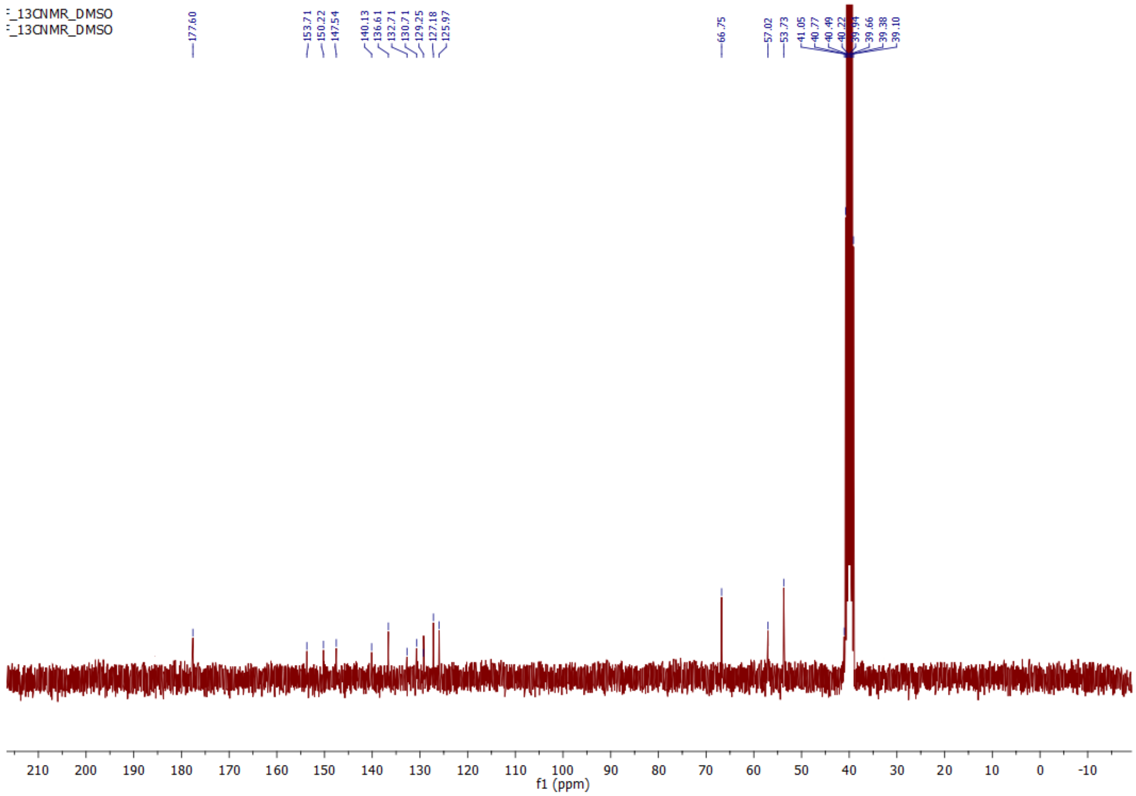

Supporting Information

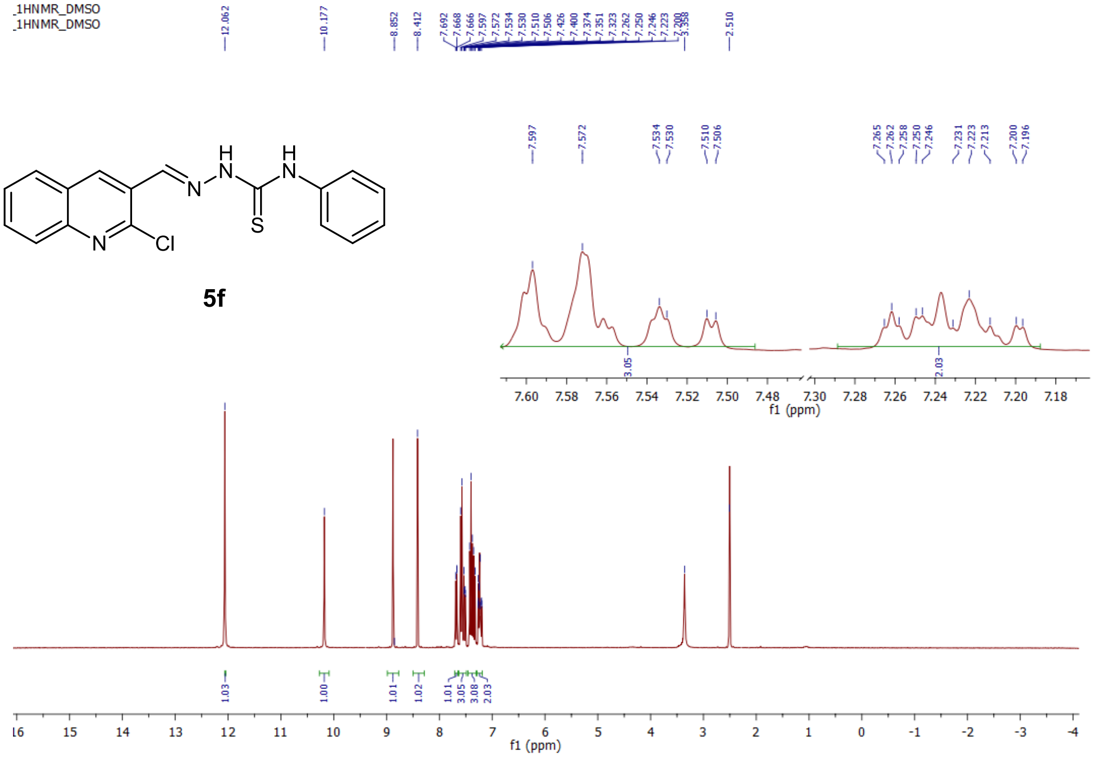

5f

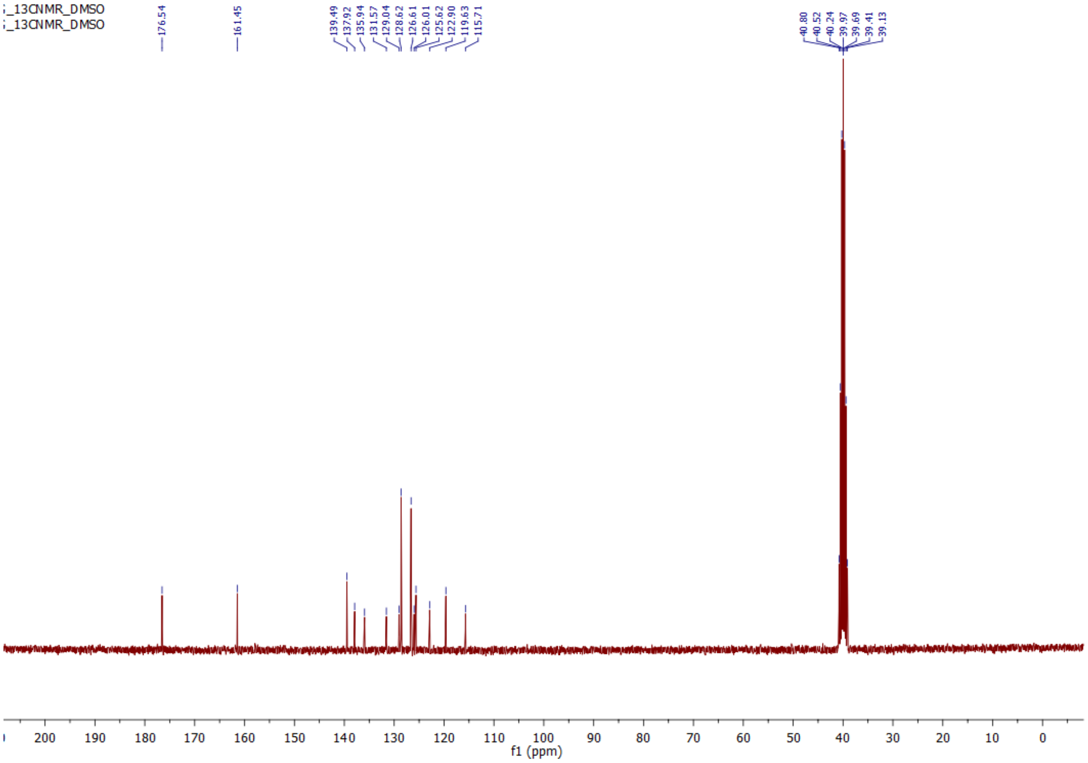

Supporting Information

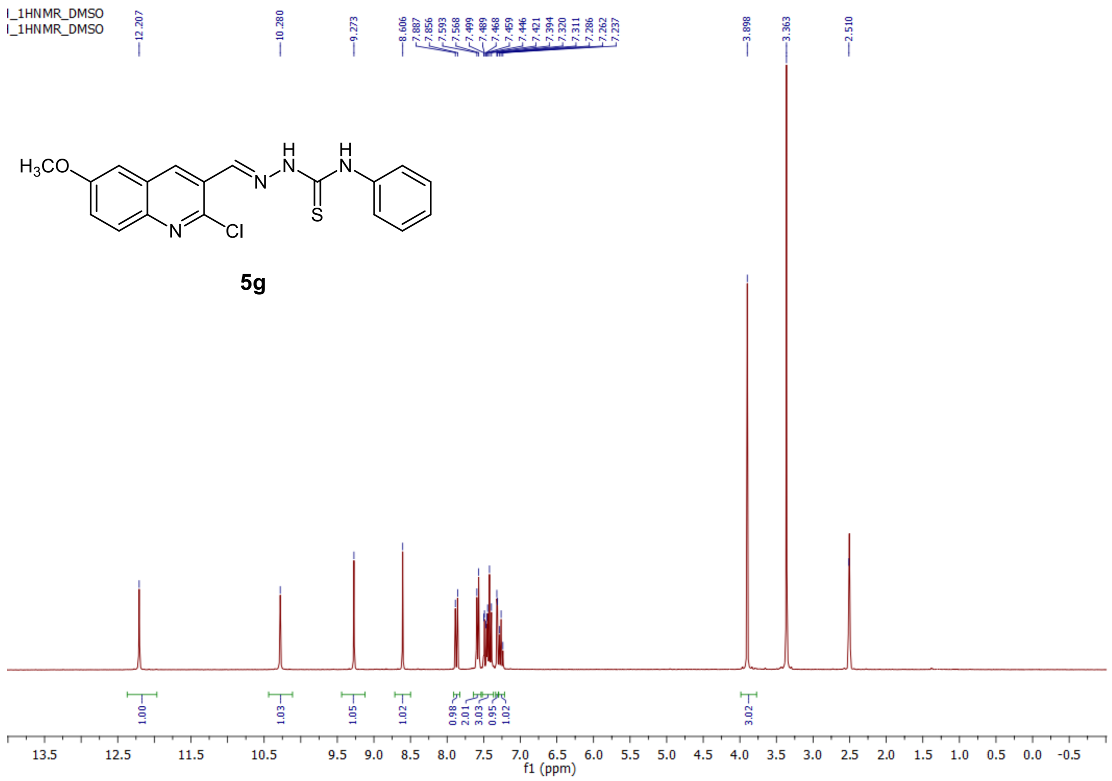

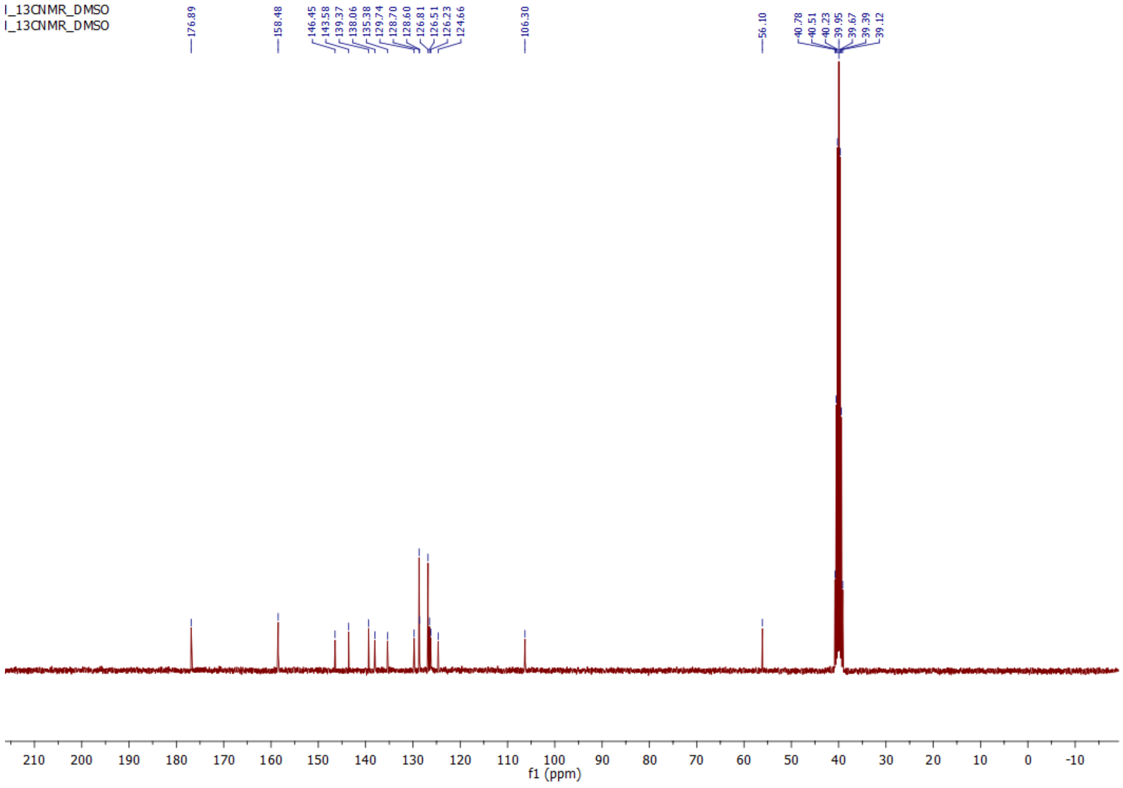

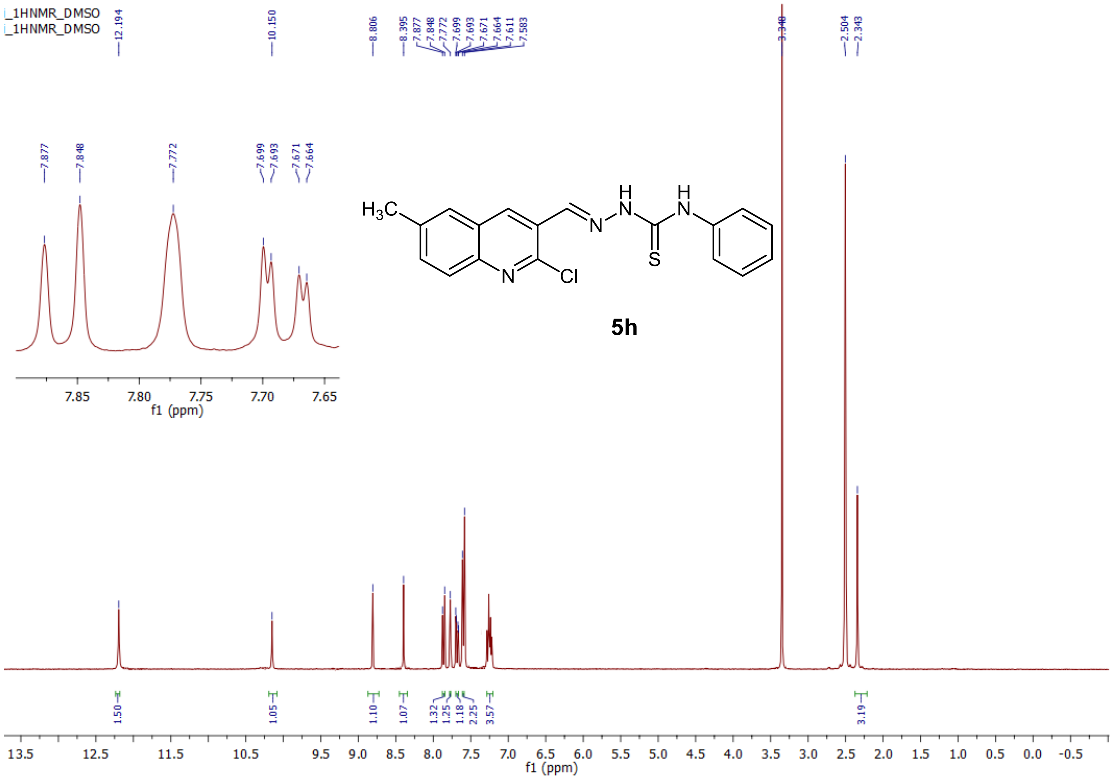
_1HNMR_DMSO
_1HNMR_DMSO
12.194
10.150
8.806
8.395
7.877
7.848
7.772
7.699
7.693
7.671
7.664
7.611
7.583
2.504
2.343
H3C
N
Cl
N
H
N
H
N
S
5h
1.50
1.05
1.10
1.07
1.32
1.25
1.18
2.25
3.57
3.19
f1 (ppm)

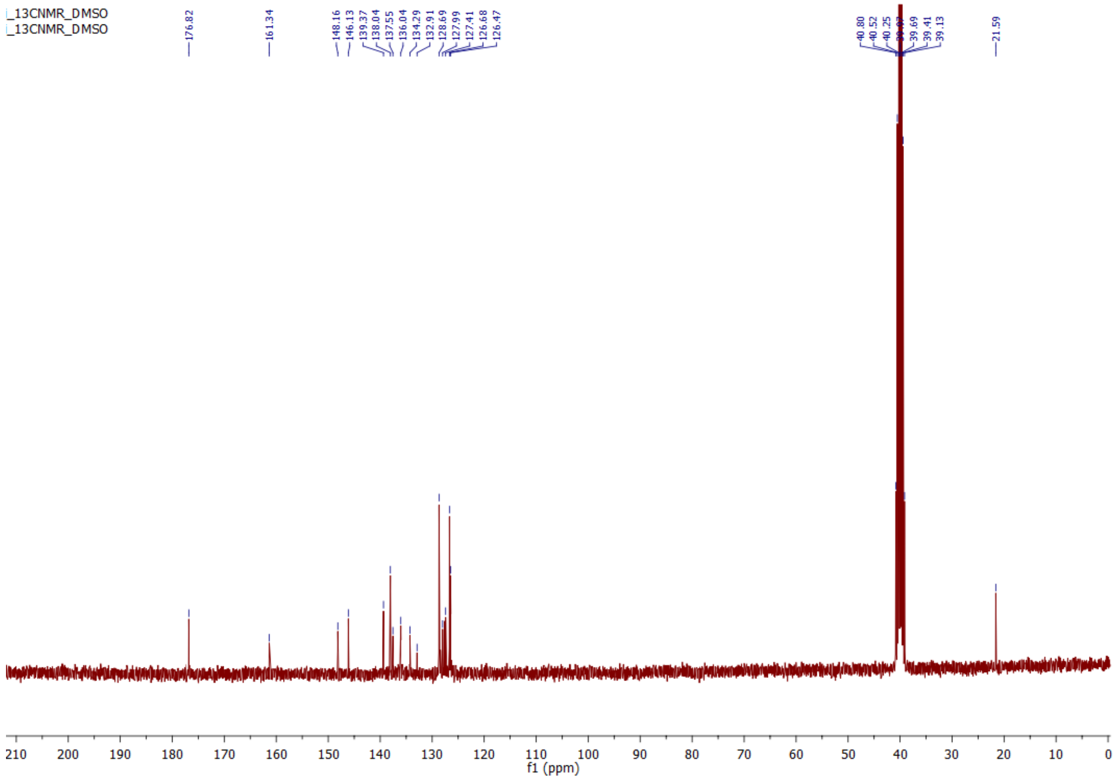
_13CNMR_DMSO
_13CNMR_DMSO
176.82
161.34
148.16
146.13
139.37
138.04
137.55
136.04
134.29
132.91
128.69
127.99
127.41
126.68
126.47
40.80
40.52
40.25
39.69
39.41
39.13
21.59
f1 (ppm)

Supporting Information

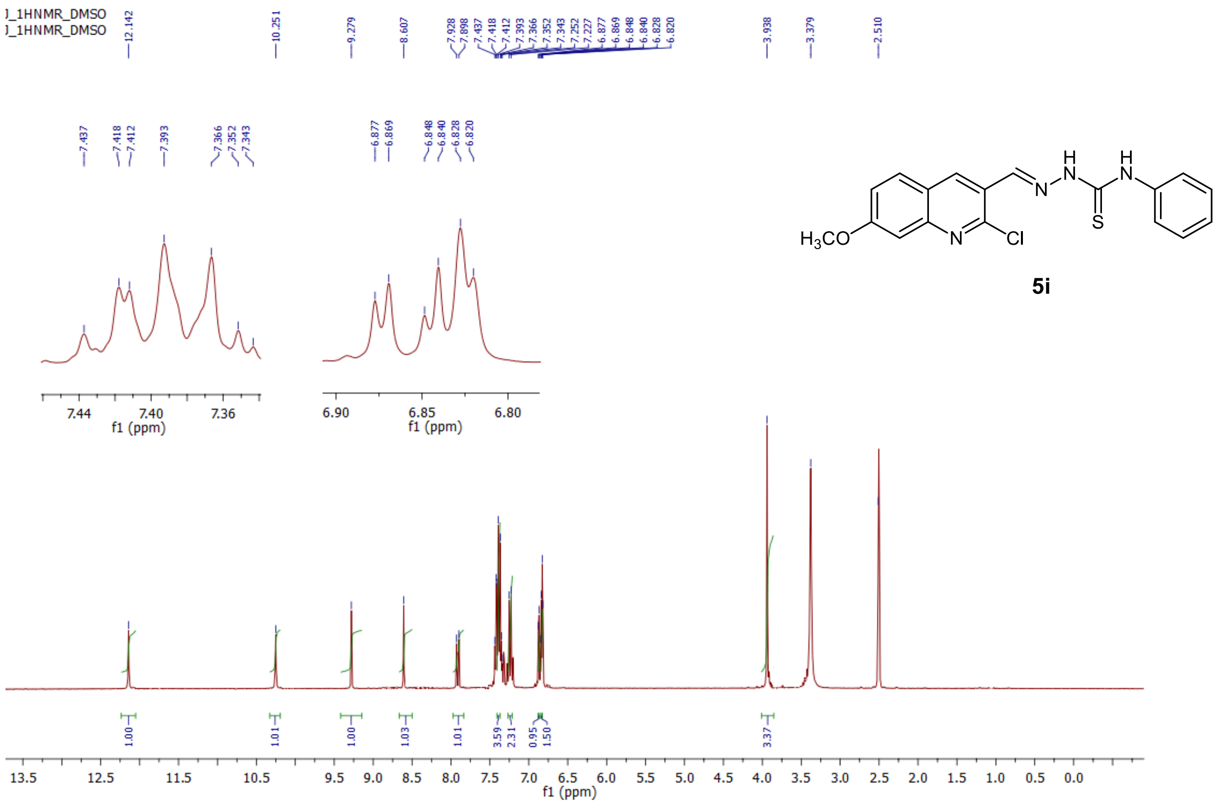

5i

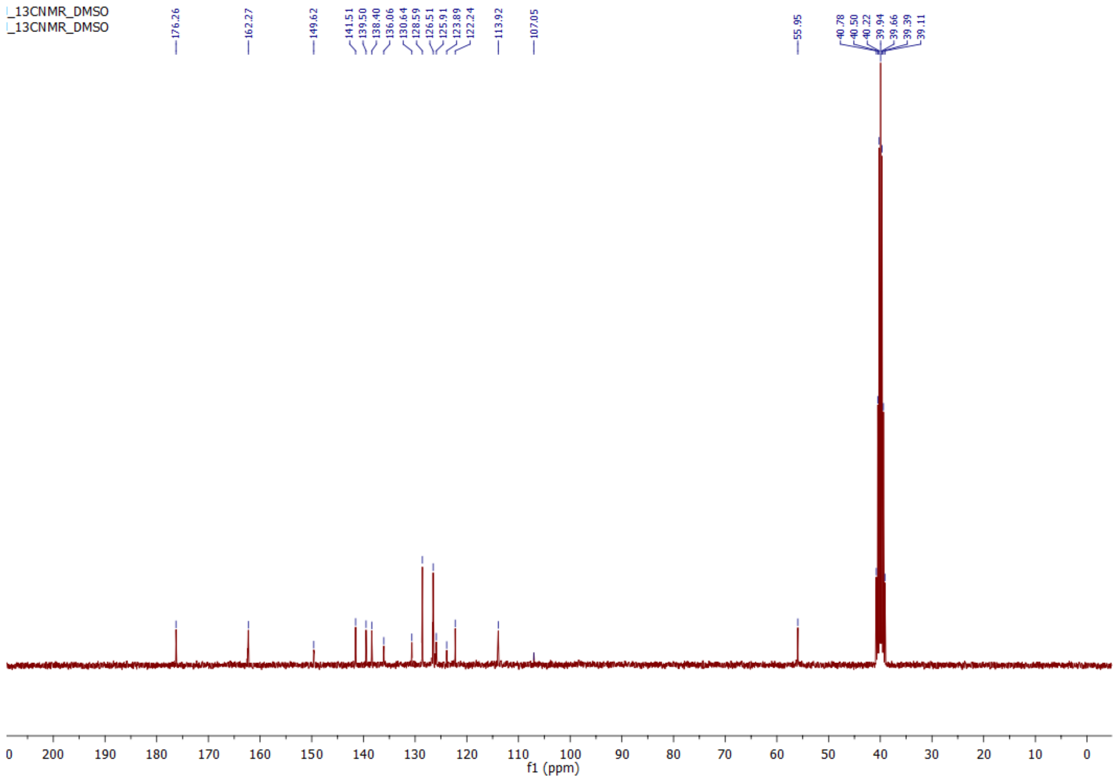

Supporting Information

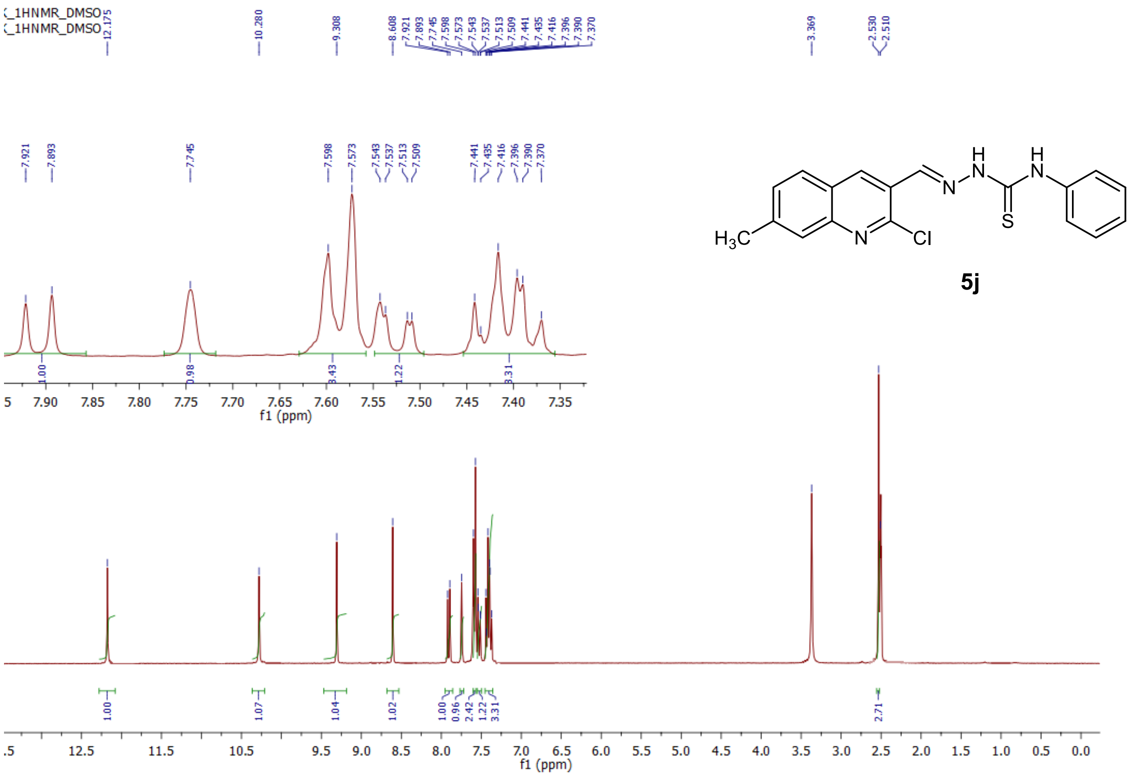

5j

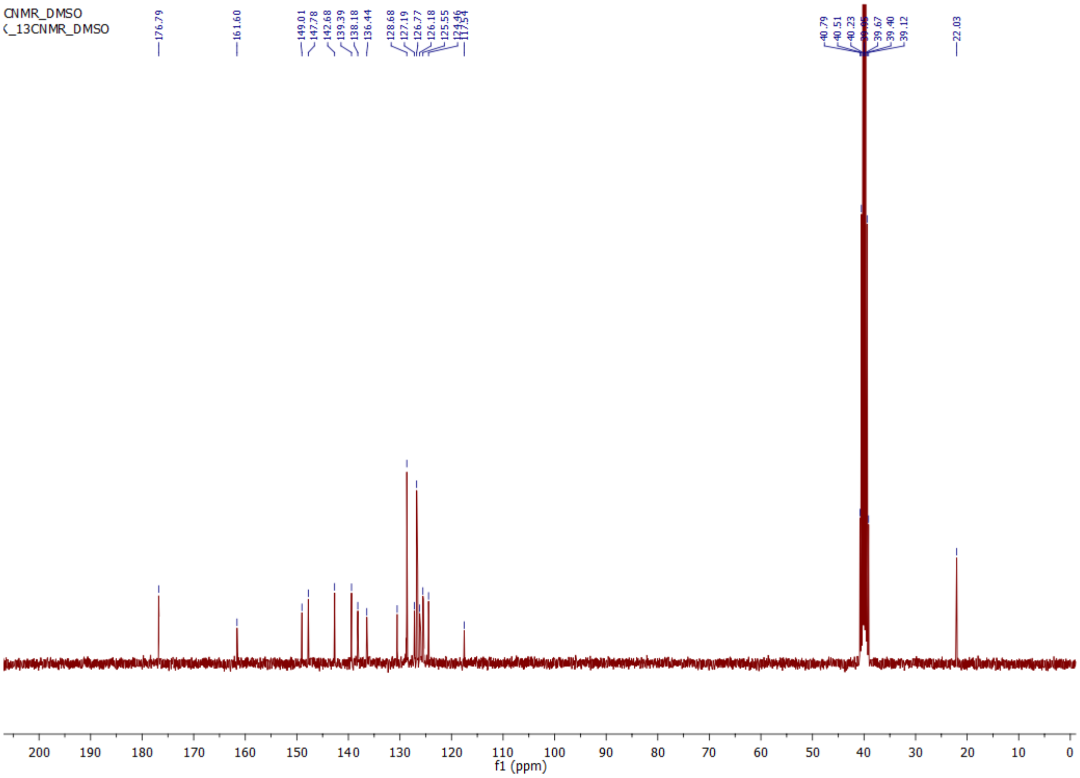

Supporting Information

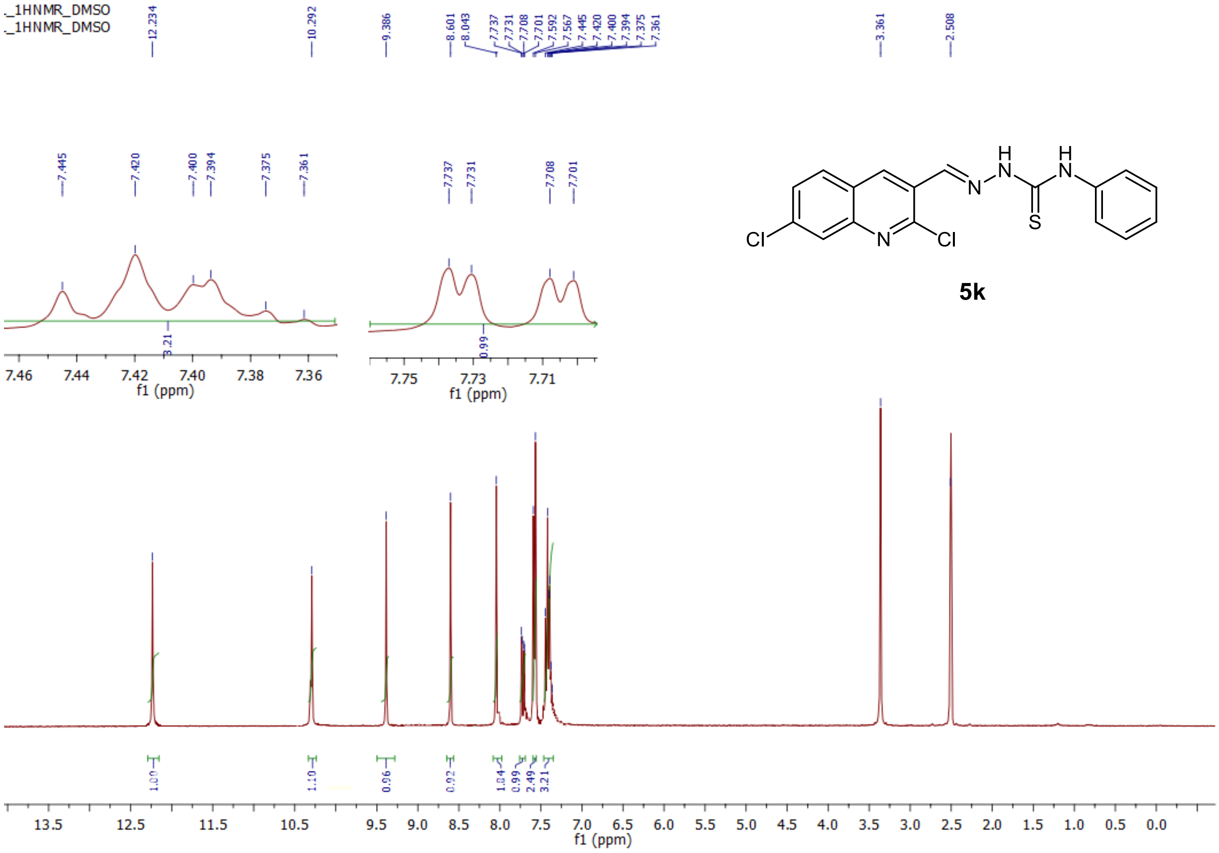

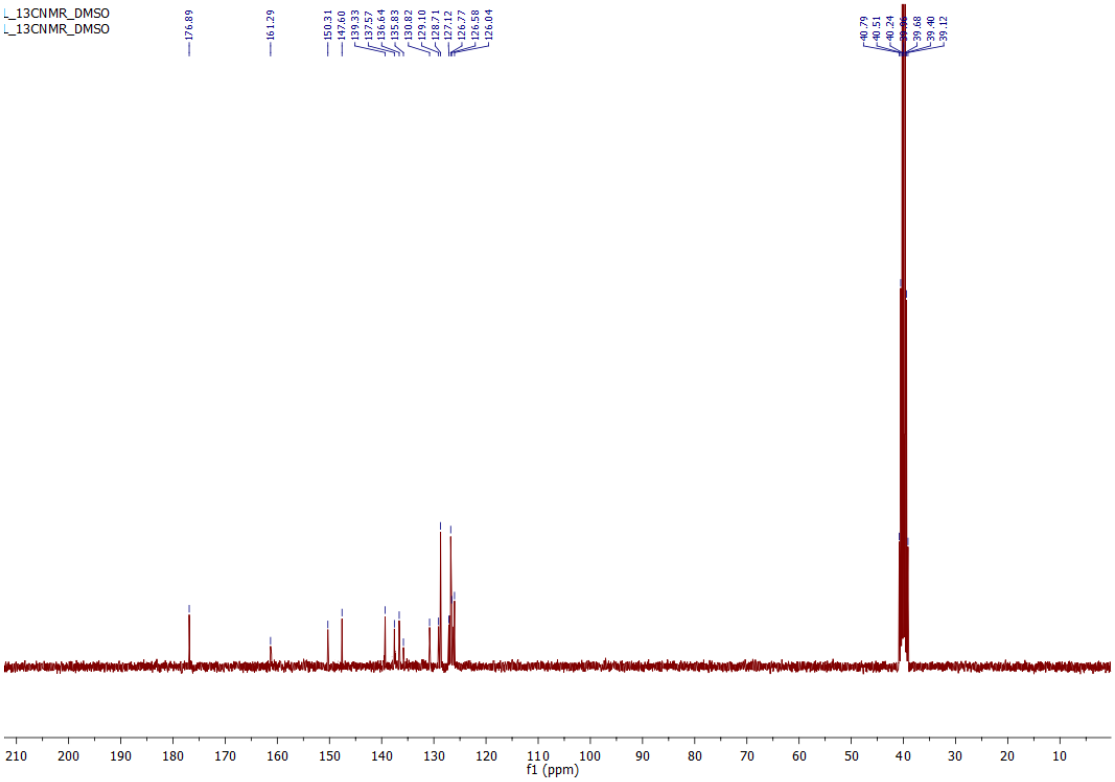

Supporting Information

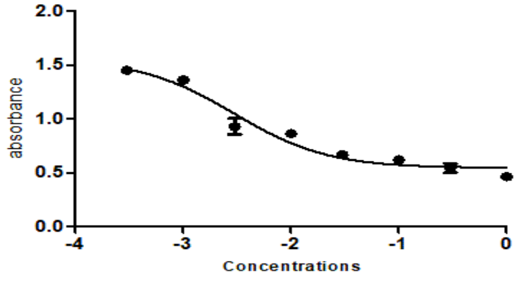

5a

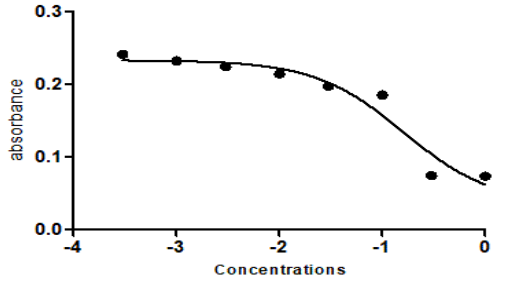

5b

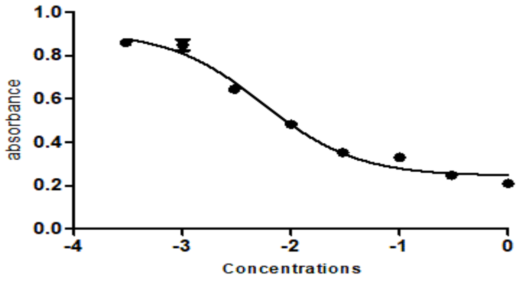

5c

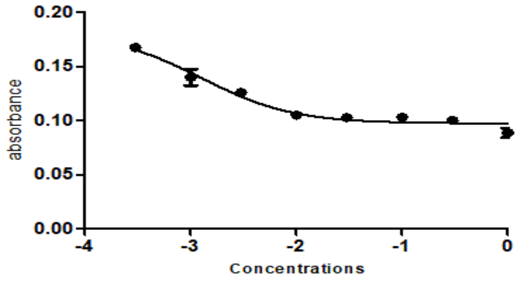

5d

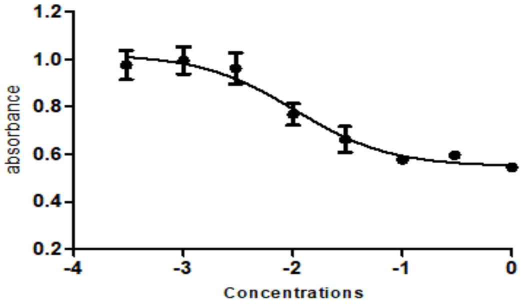

5e

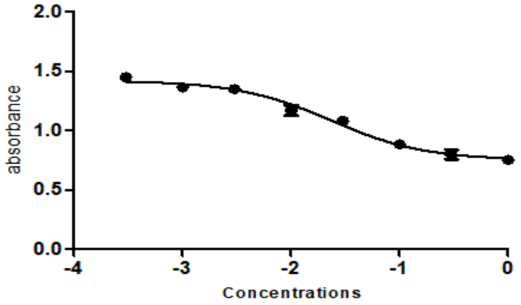

5f

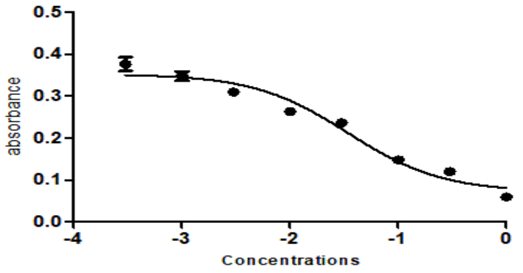

5g

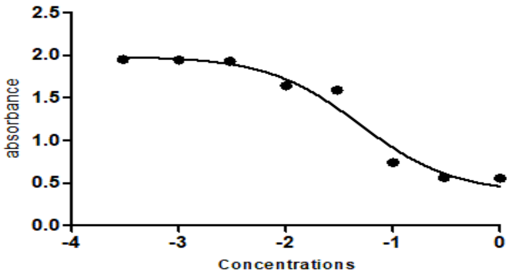

5h

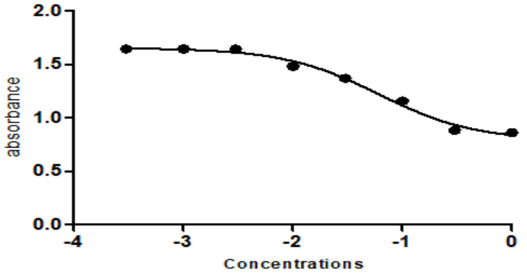

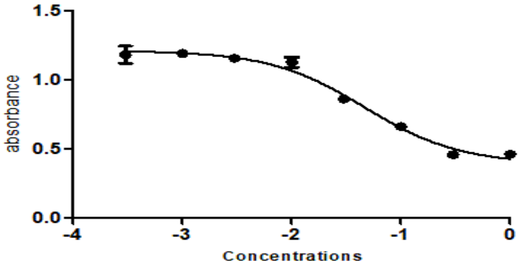

Supporting Information

5i

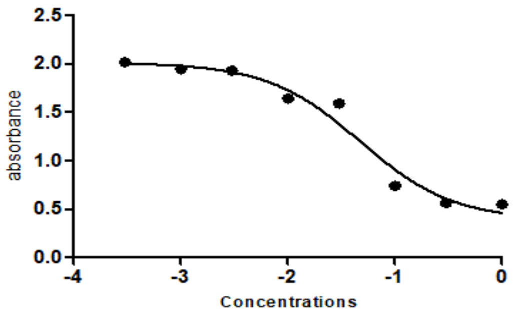

5k

5j

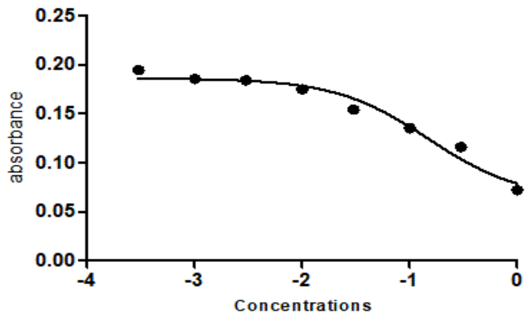

Galantamine

Figure S1. Dose response curves for enzyme inhibition activity (AChE)

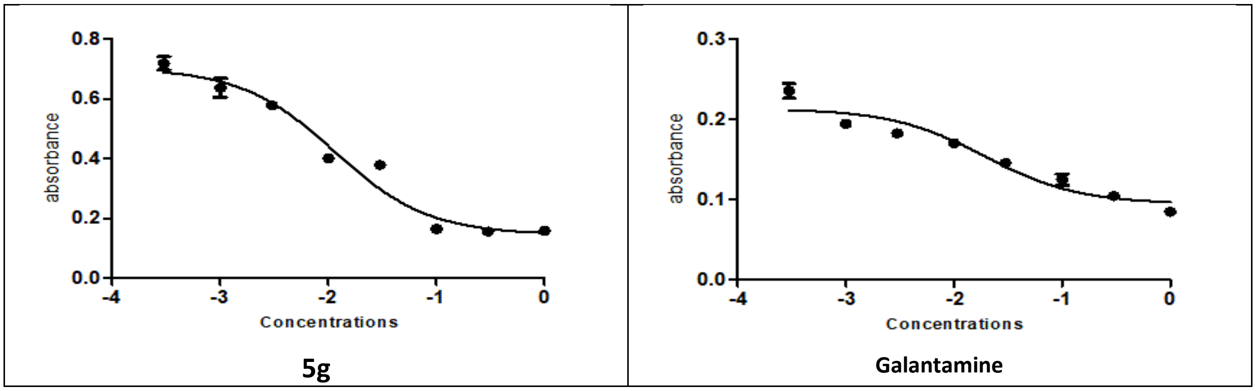

Figure S2. Dose response curves for enzyme inhibition activity (BChE)

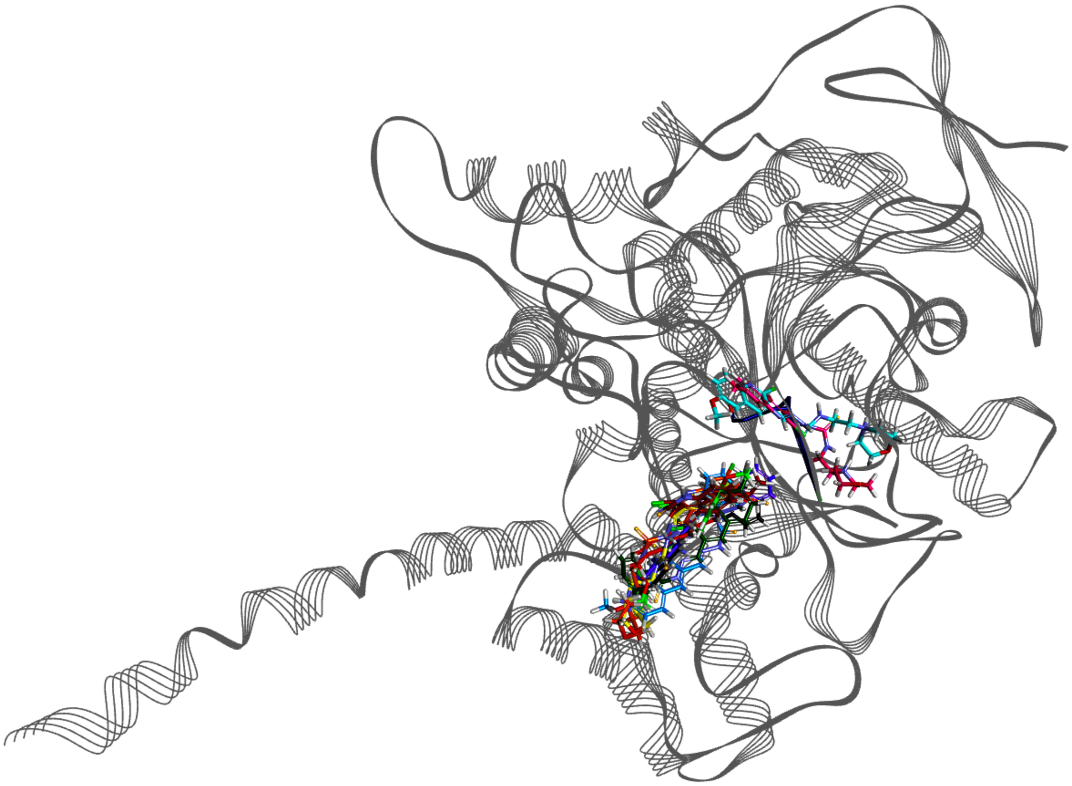

Figure S3. An overview of the active site of AChE containing cognate ligand and all the tested compounds.

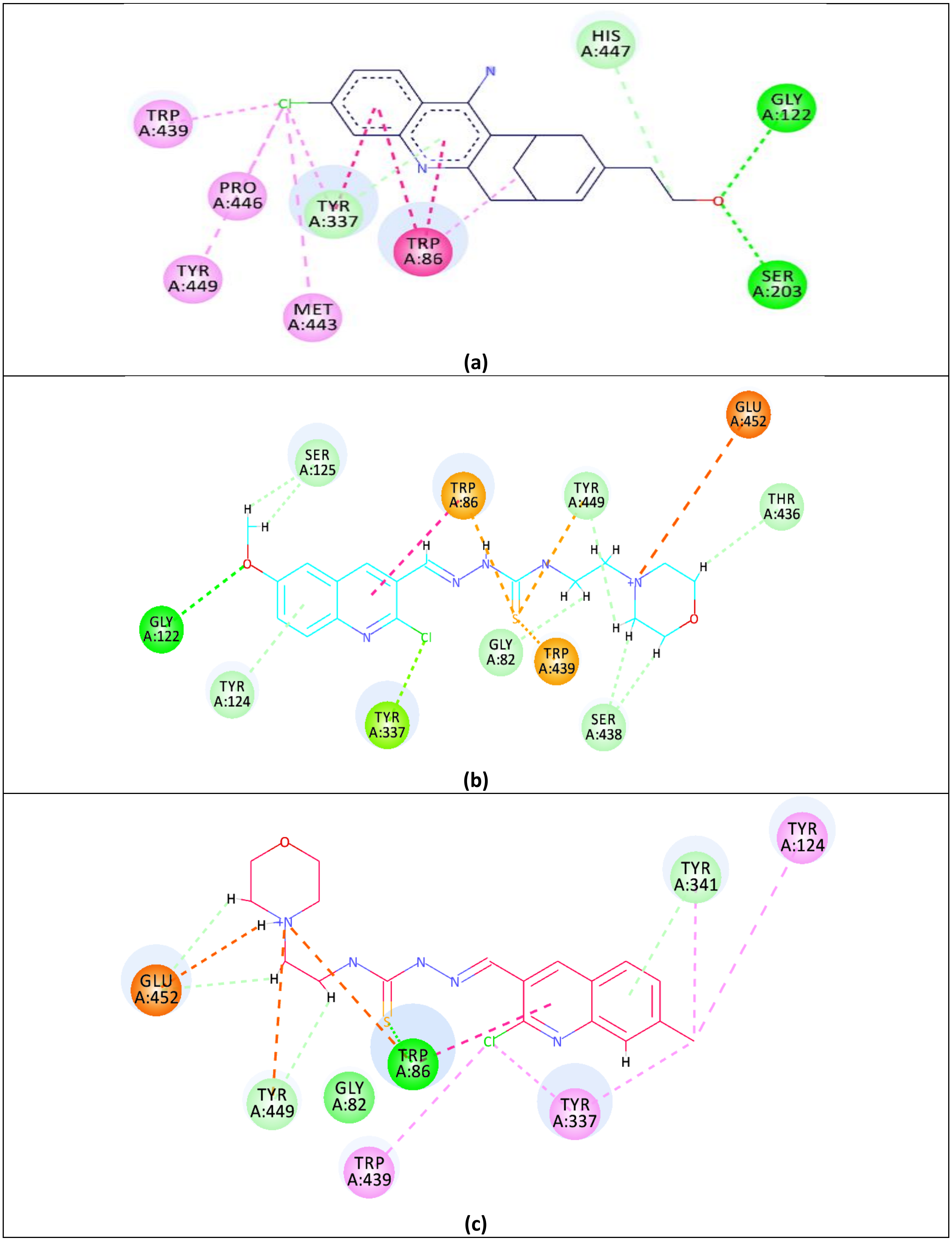

Figure S4. 2D interactions of **huprine W** (a), compounds **5b** (b) and **5d** (c) with amino acid residues.

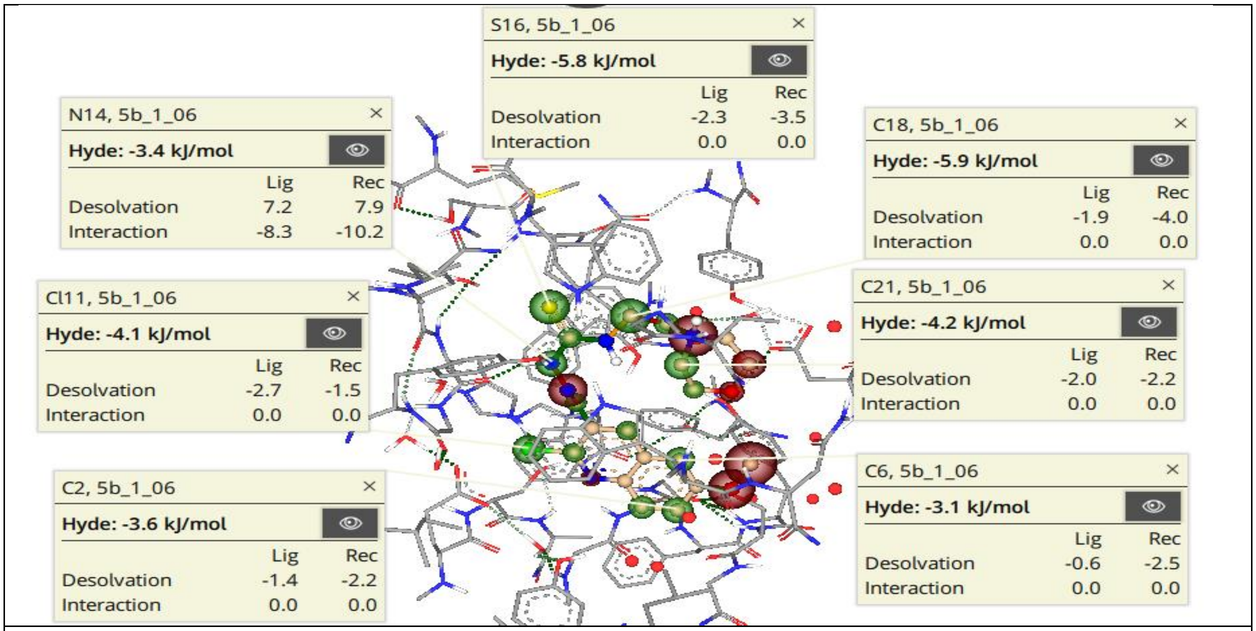

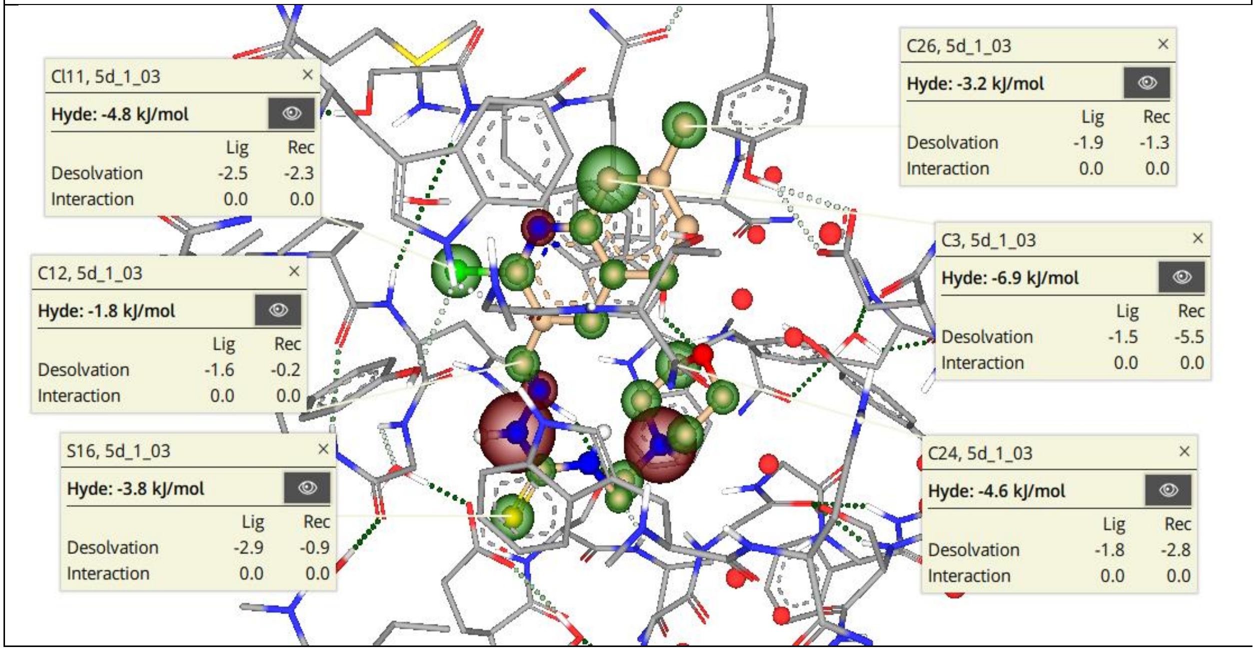

Figure S5. Visual and investigative modes of the docked pose of compounds **5b** (above) and **5d** (below) within the active site of AChE.
